# Prevalence and risk factors for suicide in patients with sepsis: nationwide cohort study in South Korea

**DOI:** 10.1192/bjo.2022.19

**Published:** 2022-03-10

**Authors:** Tak Kyu Oh, Hye Yoon Park, In-Ae Song

**Affiliations:** Department of Anesthesiology and Pain Medicine, Seoul National University Bundang Hospital, South Korea; and Department of Anesthesiology and Pain Medicine, College of Medicine, Seoul National University, South Korea; Department of Psychiatry, Seoul National University Hospital, South Korea; Department of Anesthesiology and Pain Medicine, Seoul National University Bundang Hospital, South Korea

**Keywords:** Depressive disorders, primary care, post-traumatic stress disorder, risk assessment, suicide

## Abstract

**Background:**

Although a recent study reported that survivors of critical illness have an increased risk of suicide, the suicide rate and factors associated with suicide in patients with sepsis have not yet been investigated.

**Aims:**

We aimed to examine the prevalence and risk factors of suicide among patients with sepsis in South Korea.

**Method:**

All adult patients who were admitted to all hospitals in South Korea with a main diagnosis of sepsis, from 1 January 2010 to 31 December 2018, were included in the study. The primary outcome was suicide within 1 year after sepsis diagnosis.

**Results:**

A total of 251 837 adult patients with sepsis were included, of which 132 691 patients (52.7%) died within 1 year after the diagnosis of sepsis, and death by suicide was the cause in 3903 patients (1.5%). Older age, male gender, living in a rural area, higher Charlson Comorbidity Index and Elixhauser Comorbidity Index scores, invasive treatment (continuous renal replacement therapy and mechanical ventilator support) and admission to a hospital with low annual case volumes were associated with a higher risk of suicide. In addition, concurrent substance misuse, post-traumatic stress disorder, bipolar disorder, dementia and previous attempt of suicide or self-harm were associated with a higher risk of suicide.

**Conclusions:**

During the 1-year follow-up period, 1.5% of patients died by suicide after the diagnosis of sepsis in South Korea. Knowledge of the factors associated with suicide might allow for earlier intervention to potentially reduce the number of suicide attempts in patients with sepsis.

Sepsis is a life-threatening condition that develops because of dysregulation of the host immune response in response to infection.^1^ In South Korea, the incidence of sepsis increased from 173.8 per 100 000 population in 2007 to 233.6 per 100 000 population in 2016, whereas the hospital mortality decreased from 30.9% in 2007 to 22.6% in 2016.^2^ Globally, an estimated 48.9 million cases of sepsis and 11.0 million sepsis-related deaths were recorded in 2017, suggesting that sepsis is a major health problem worldwide.^3^

Suicide is a major public health issue, accounting for 1.3% of all deaths worldwide in 2019.^4^ In South Korea, suicide is a serious public health issue; the prevalence of suicide was 24.6 per 100 000 persons in 2019, which was the highest among the Organization for Economic Cooperation and Development countries.^5^ Moreover, the prevalence of death from suicide was 5.26% of total deaths (84 934 deaths by suicide/1 615 288 total deaths) from 2011 to 2016 in South Korea.^6^ Suicide might occur in patients with sepsis with several risk factors, worsening conditions, disabilities and psychological deficits that cause patients with sepsis to suffer so much that they no longer not want to live.^7^ Moreover, patients with sepsis are known to suffer from post-sepsis syndrome, which is characterised by neurocognitive impairment, functional disability, psychological deficits and worsening medical conditions.^8^ These worsening conditions after diagnosis of sepsis could increase the rate of suicide mortality among patients with sepsis. Similarly, a recent cohort study by Fernando et al^9^ reported that 0.2% of intensive care unit (ICU) survivors of critical illness died by suicide in the province of Ontario in Canada. They suggested that pre-existing psychiatric illness and receipt of invasive life support were significant risk factors for suicide among ICU survivors.^9^ Lund-Sorensen et al also reported a significant relationship between hospital admission with infection and increased risk of death by suicide among a Danish cohort.^10^ This relationship might have occurred because of a biological mechanism – an inflammatory reaction resulting from infection increases risk of suicidal behavior.^10,11^ However, in addition to infection, the information regarding the prevalence of suicide and associated factors in patients with sepsis is still lacking. Considering the global burden of sepsis among critically ill patients,^3^ better understanding of the prevalence and associated risk factors for suicide mortality in patients with sepsis is currently needed.

Therefore, using a nationwide cohort database in South Korea, we aimed to investigate the prevalence of suicide and associated factors in patients with sepsis. We included various factors that could be potentially related to the risk of suicide, such as physical characteristics, information related to socioeconomic status, treatment information, medical condition and concurrent psychiatric disorders.

## Method

### Study design

In this population-based cohort study of a nationwide cohort in South Korea, we followed the Strengthening the Reporting of Observational Studies in Epidemiology guidelines. The study protocol was approved by the Institutional Review Board of Seoul National University Bundang Hospital (approval number X-1912-580-902), and the National Health Insurance Service (NHIS) permitted data-sharing after approval of the study protocol (approval number NHIS-2020-1-095). The requirement for informed consent was waived because we performed the data analysis retrospectively in an anonymised manner.

### Database (NHIS and Statistics Korea)

The NHIS database was used for this study. In South Korea, all disease diagnoses and prescription information regarding drugs and/or procedures have to be registered in the NHIS database as the sole public health insurance system. These registrations enable patients to receive government financial support for treatment expenses. The disease diagnoses are registered in the NHIS database, using ICD-10 codes. In addition, we used the Statistics Korea database to extract the death date and main cause of death, up to 31 December 2019. Physicians are required to register the main cause of death (primary disease) in the Statistics Korea database in South Korea. First, we obtained approval from the NHIS for data extraction from the NHIS database, which contains demographic data, socioeconomic status, disease diagnoses and treatment information of the study population. Next, we obtained approval from Statistics Korea for data-sharing regarding death date and main causes of death among the study population. These two databases were linked by the ‘resident registration number’, a mandatory identification number issued to residents of South Korea regardless of nationality. The linkage process was performed cooperatively by medical record technicians in the two centres (NHIS and Statistics Korea).

### Patients with sepsis

We included all adult patients (≥18 years of age) who were admitted to all hospitals in South Korea from 1 January 2010 to 31 December 2018, with a main diagnosis of sepsis (ICD-10 codes A40, A41 and R65.2) based on the ICD-10 codes. The main diagnosis was determined by the NHIS for each patient after hospital discharge or death, as the disease that required the greatest treatment or examination during the patient's hospital stay. If a patient was admitted to the hospital twice or more with the main diagnosis of sepsis during the study period, only the first episode of hospital admission was considered in this study. In addition, cases with missing date of death were excluded from the analysis because we focused on mortality among patients with sepsis.

### Primary outcome

The primary outcome of this study was suicide within 1 year after the diagnosis of sepsis among patients with sepsis. Suicide was defined as death by suicide attempt or self-harm (ICD-10 codes X60–X84 and Y870), in accordance with a previous study.^9^ Patients who died from suicide were included in the suicide group, whereas patients who died from other causes were included in the non-suicide death group. If patients with sepsis survived over 1 year after diagnosis of sepsis, they were considered as survivors.

### Covariates

Physical characteristics, socioeconomic status, treatment, medical conditions and concurrent psychiatric disorders were considered as covariates because they might influence risk of death by suicide.^12^ Age and gender were the physical covariates. Residence at the time of diagnosis of sepsis and household income level were collected to reflect the socioeconomic status of the patients with sepsis. All patients were classified into two groups according to the residence: urban area (Seoul and other metropolitan cities) and other areas. Classification was based on home postal codes collected upon hospital admission. These data were collected because there might be inequality in the utilisation of health services in rural versus urban areas.^13^ Household income level was extracted using insurance premium of all patients as a covariate; it was classified into four groups, using quartile ratios (quartiles 1–4, lowest to highest). All individuals in South Korea are registered in the NHIS^14^ and are divided into two groups: employee insured and self-employed insured. The insurance premium for employee-insured individuals was determined according to the income, whereas the insurance premium for self-employed-insured individuals was determined according to the income, property value, living standards and rate of participation in economic activities. To reflect the comorbid status of all patients with sepsis, the Charlson Comorbidity Index (CCI) and Elixhauser Comorbidity Index (ECI) were calculated using ICD-10 codes within 1 year before the diagnosis of sepsis, as shown in Supplementary Tables 1 and 2 available at https://doi.org/10.1192/bjo.2022.19.^15^ The annual case volume of hospital admissions for sepsis in South Korea during 2010–2018 was calculated and considered as a covariate. All patients were classified into four groups according to the annual case volume, using the quartile ratio, based on each hospital in which patients with sepsis were admitted: quartile 1, ≤ 235; quartile 2, 236 to < 710; quartile 3, 710 to < 1743 and quartile 4, ≥ 1743. The admitting department (medical or surgical department) for all of the patients with sepsis was also collected as a covariate. For treatment information, continuous renal replacement therapy (CRRT) use, vasopressor use, extracorporeal membrane oxygenation support and mechanical ventilator support were collected as covariates. Total number of hospital admissions with a main diagnosis of sepsis during the study period was also collected and divided into categorical variables (1, 2–3, 4–5, 6–7 and ≥8). Moreover, ICU admissions with the main diagnosis of sepsis were also collected and considered as a covariate. As mental disorders are well-known risk factors for suicide,^16^ concurrent psychiatric illnesses such as depression (ICD-10 codes F32, F33, F34.1), anxiety disorder (ICD-10 codes F40, F41), substance misuse (ICD-10 codes F10–F19), post-traumatic stress disorder (PTSD) (ICD-10 code F43.1), bipolar disorder (ICD-10 code F31), schizophrenia or schizophrenic affective disorder (ICD-10 codes F20, F25), and dementia (ICD-10 codes F00–F03, F05.1, G30–G31) were also included. In addition, history of previous suicide attempt or self-harm (ICD-10 codes X60–X84 and Y870) was extracted using ICD-10 codes within 1 year before and after the diagnosis of sepsis.

### Statistical analysis

The clinicopathological characteristics of the patients with sepsis were presented as median values with interquartile ranges (IQRs) for continuous variables, because the distribution of continuous variables (age, CCI and ECI) was not normally distributed according to Kolmogorov–Smirnov tests. The categorical variables were presented as numbers with percentages. To compare the clinicopathological characteristics between the three groups (suicide group, non-suicide death group and survivors), we used the Kruskal–Wallis test and chi-squared test for continuous variables and categorical variables, respectively. The proportion of extracorporeal membrane oxygenation support among patients with sepsis was compared with Fisher's exact test.

We performed competing risk analysis by using the Fine and Gray method to examine associated factors for mortality by suicide and non-suicide mortality,^17^ because the Fine and Gray method can be used to assess the deaths from other causes (non-suicide mortality). For example, if a patient died from other causes, they would not die because of suicide, and the death from another cause should be handled as a competing risk. All covariates were included in the Fine and Gray model for adjustment, and results were presented as subdistribution hazard ratios (sHR) with 95% confidence intervals. Schoenfeld-type residuals were used to assess the proportional subdistribution hazard assumption of the Fine and Gray models, and no multicollinearity was identified between the included variables with a variance inflation factor <2.0. In addition, we tested interactions between the variables for suicide mortality, and there was no important interaction between variables that should be considered. Next, we performed subgroup analysis with the Fine and Gray model for suicide mortality according to gender, and the total cohort was divided into a male group and female group. We also performed three sensitivity analyses. First, after excluding patients with sepsis who had concurrent psychiatric disorders or a history of self-harm or suicidal attempt, the Fine and Gray model for suicide mortality was constructed. Second, we fitted the Fine and Gray model for suicide mortality in patients with sepsis who were admitted to ICUs. Finally, the cumulative incidence of suicide mortality according to predictors identified in the Fine and Gray model were applied to the total study population. All statistical analyses were performed with R software (Windows version 4.0.3 with R packages, R Project for Statistical Computing, Vienna, Austria; see https://cran.r-project.org/mirrors.html). Specifically, ‘riskRegression,’ ‘survival’ and ‘cmprsk’ packages were used for main competing risk analyses, and statistical significance was set at *P* < 0.05.

## Results

### Study population

**Fig. 1:**
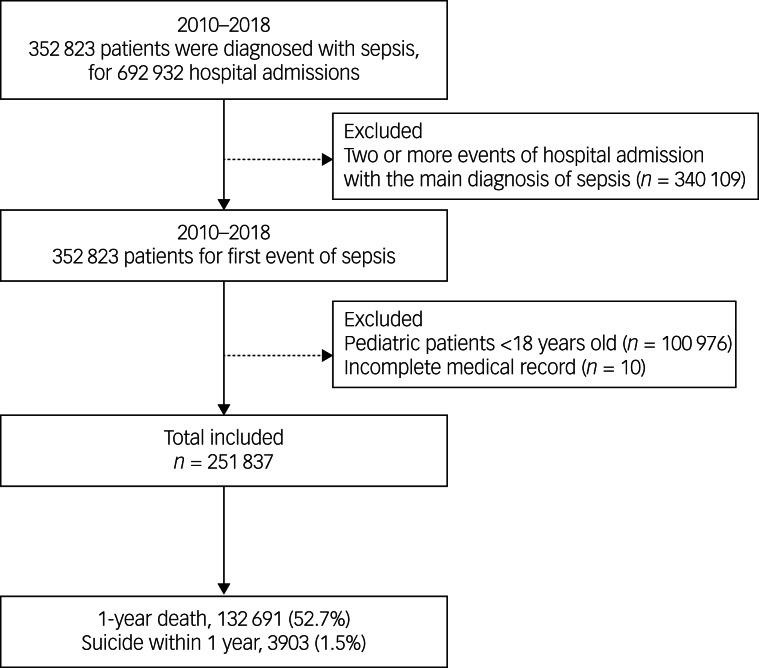
Flow chart depicting the selection process of patients with sepsis.

From 1 January 2010 to 31 December 2018, there were 692 932 hospital admissions of 352 823 patients with a main diagnosis of sepsis. The first episode of hospital admission for each patient was included in this study. After excluding 100 976 paediatric patients under 18 years of age and ten cases with incomplete medical records regarding the death date, 251 837 adult patients with sepsis were included in this study. Among them, 132 691 (52.7%) patients died within 1 year after the diagnosis of sepsis, and death by suicide was the cause in 3903 (1.5%) patients, as shown in Figure 1. Among the suicide group, 1512 (0.6%) patients with sepsis died by suicide during their hospital stay. Table 1 shows the clinicopathological characteristics of all patients with sepsis. The median age was 77 years (IQR 64–84 years), and 109 943 (43.7%) patients were male. There were 11 467 (4.6%) patients with substance misuse, and among these patients, the proportion of alcohol misuse was highest (10 194, 4.0%), followed by sedative misuse (479, 0.2%). The median values of the CCI and ECI were 6.8 (s.d. 4.5) and 18.5 (s.d. 13.3), respectively. The 30-day and 90-day mortality occurred in 75 736 (30.1%) and 102 560 (40.7%) patients, respectively. Table 2 shows the results of the comparison between the three groups (suicide group, non-suicide death group and survivors). The median age of patients in the suicide group and non-suicide group was 79.0 years (IQR 69.0–86.0 years) and 80.0 years (IQR 73.0–86.0 years), respectively, whereas that of patients in the survivor group was 71.0 years (IQR 53.0–83.0 years).

**Table 1:** Clinicopathological characteristics of the total cohort of 251 837 patients with sepsis

Variable	*N* (%)	Median [IQR]
Age, years		77 [64–84]
Gender, male	109 943 (43.7)	
Residence at diagnosis of sepsis		
Urban area	85 071 (33.8)	
Rural area	166 766 (66.2)	
Income level at diagnosis of sepsis		
Quartile 1 (lowest)	45 920 (18.2)	
Quartile 2	33 901 (13.5)	
Quartile 3	45 881 (18.2)	
Quartile 4 (highest)	84 028 (33.4)	
Unknown	42 107 (16.7)	
Charlson Comorbidity Index		6 [3–9]
Elixhauser Comorbidity Index		17 [8–28]
Admitting department with main diagnosis of sepsis		
Medical department	224 278 (89.1)	
Surgical department	27 559 (10.9)	
Annual case volume of sepsis treatment		
Quartile 1, ≤ 235	74 720 (29.7)	
Quartile 2, 236 to < 710	78 023 (31.0)	
Quartile 3, 710 to < 1743	59 804 (23.7)	
Quartile 4, ≥ 1743	39 290 (15.6)	
CRRT use	6954 (2.8)	
Vasopressor use	43 787 (17.4)	
ECMO support	263 (0.1)	
Mechanical ventilator support	34 572 (13.7)	
ICU admission	60 165 (23.9)	
Total number of hospital admissions for sepsis		
1	149 520 (59.4)	
2–3	73 798 (29.3)	
4–5	16 157 (6.4)	
6–7	5648 (2.2)	
≥8	6714 (2.7)	
Concurrent psychiatric illness		
Depression	72 344 (28.7)	
Anxiety disorder	74 731 (29.7)	
Substance misuse^a^	11 467 (4.6)	
PTSD	100 (0.0)	
Bipolar disorder	35 225 (14.0)	
Schizophrenia or schizophrenic affective disorder	11 738 (4.7)	
Dementia	104 175 (41.4)	
History of self-harm or suicidal attempt	81 (0.0)	
Year of diagnosis of sepsis		
2010	27 849 (11.1)	
2011	23 342 (9.3)	
2012	25 057 (9.9)	
2013	25 334 (10.1)	
2014	27 453 (10.9)	
2015	28 711 (11.4)	
2016	29 786 (11.8)	
2017	30 858 (12.3)	
2018	33 447 (13.3)	
Hospital mortality	52 970 (21.0)	
Death by suicide during hospital admission	1512 (0.6)	
30-day mortality	75 736 (30.1)	
90-day mortality	102 560 (40.7)	
1-year mortality	132 691 (52.7)	

IQR, interquartile range; CRRT, continuous renal replacement therapy; ECMO, extracorporeal membrane oxygenation; ICU, intensive care unit; PTSD, post-traumatic stress disorder.

a. Alcohol, 10 194 (4.0%); opioids, 67 (0.0%); cannabis, 13 (0.0%); sedatives, 479 (0.2%); cocaine, 12 (0.0%); stimulants, 19 (0.0%), hallucinogens, 9 (0.0%); nicotine, 150 (0.1%); inhalants, 22 (0.0%) and other psychotic agents, 502 (0.2%).

**Table 2:** Comparison of the clinicopathological characteristics between the three groups (suicide group, non-suicide death group and survivors)

Variable	Suicide group, *n* = 3903	Non-suicide death group, *n* = 128 788	Survivors, *n* = 119 146	*P*-value
Age, years	79.0 [69.0–86.0]	80.0 [73.0–86.0]	71.0 [53.0–80.0]	<0.001
Gender, male	2079 (53.3)	60 078 (46.6)	47 786 (40.1)	<0.001
Residence at diagnosis of sepsis				<0.001
Urban	1126 (28.8)	40 955 (31.8)	43 054 (36.1)	
Rural	2777 (71.2)	87 833 (68.2)	76 092 (63.9)	
Income level at diagnosis of sepsis				<0.001
Quartile 1 (lowest)	725 (18.6)	23 757 (18.4)	21 438 (18.0)	
Quartile 2	502 (12.9)	16 045 (12.5)	17 354 (14.6)	
Quartile 3	703 (18.0)	21 768 (16.9)	23 410 (19.6)	
Quartile 4 (highest)	1263 (32.4)	42 840 (33.3)	39 925 (33.5)	
Unknown	710 (18.2)	24 378 (18.9)	17 019 (14.3)	
Charlson Comorbidity Index	6 [4–9]	7 [4–11]	5 [2–8]	<0.001
Elixhauser Comorbidity Index	19 [10–29]	21 [11–30]	14 [4–24]	<0.001
Admitting department				<0.001
Medical department	3131 (80.2)	112 813 (87.6)	108 334 (90.9)	
Surgical department	772 (19.8)	15 975 (12.4)	10 812 (9.1)	
Total case volume of sepsis treatment				<0.001
Quartile 1, ≤ 235	1321 (33.8)	43 432 (33.7)	29 967 (25.2)	
Quartile 2, 236 to < 710	1405 (36.0)	45 276 (35.2)	31 342 (26.3)	
Quartile 3, 710 to < 1743	940 (24.1)	30 742 (23.9)	28 122 (23.6)	
Quartile 4, ≥ 1743	237 (6.1)	9338 (7.3)	29 715 (24.9)	
CRRT use	168 (4.3)	5591 (4.3)	1195 (1.0)	<0.001
Vasopressor use	849 (21.8)	30 934 (24.0)	12 004 (10.1)	<0.001
ECMO support	9 (0.2)	217 (0.2)	37 (0.0)	<0.001
Mechanical ventilator support	988 (25.3)	28 799 (22.4)	4785 (4.0)	<0.001
ICU admission	1166 (29.9)	38 759 (30.1)	20 240 (17.0)	<0.001
Total number of hospital admissions for sepsis				<0.001
1	2434 (62.4)	83 851 (65.1)	63 235 (53.1)	
2–3	1124 (28.8)	35 869 (27.9)	36 805 (30.9)	
4–5	222 (5.7)	6157 (4.8)	9778 (8.2)	
6–7	65 (1.7)	1729 (1.3)	3854 (3.2)	
≥8	58 (1.5)	1182 (0.9)	5474 (4.6)	
Concurrent psychiatric illness				
Depression	1136 (29.1)	36 146 (28.1)	35 062 (29.4)	<0.001
Anxiety disorder	1140 (29.2)	36 281 (28.2)	37 310 (31.3)	<0.001
Substance misuse	241 (6.2)	5428 (4.2)	5798 (4.9)	<0.001
PTSD	5 (0.1)	28 (0.0)	67 (0.1)	<0.001
Bipolar disorder	633 (16.2)	19 058 (14.8)	15 534 (13.0)	<0.001
Schizophrenia or schizophrenic affective disorder	205 (5.3)	6701 (5.2)	4832 (4.1)	<0.001
Dementia	1992 (51.0)	72 393 (56.2)	29 790 (25.0)	<0.001
History of self-harm or suicidal attempt	11 (0.3)	35 (0.0)	35 (0.0)	<0.001
Year of diagnosis of sepsis				<0.001
2010	467 (12.0)	14 795 (11.5)	12 587 (10.6)	
2011	355 (9.1)	12 782 (9.9)	10 205 (8.6)	
2012	470 (12.0)	13 629 (10.6)	10 958 (9.2)	
2013	466 (11.9)	13 432 (10.4)	11 436 (9.6)	
2014	450 (11.5)	13 776 (10.7)	13 227 (11.1)	
2015	419 (10.7)	13 978 (10.9)	14 314 (12.0)	
2016	409 (10.5)	14 166 (11.0)	15 211 (12.8)	
2017	432 (11.1)	15 251 (11.8)	15 175 (12.7)	
2018	435 (11.1)	16 979 (13.2)	16 033 (13.5)	

Data are presented as median values with interquartile ranges for continuous variables, and numbers with percentages for categorical variables. The Kruskal–Wallis test and chi-squared test were used for continuous variables and categorical variables, respectively. CRRT, continuous renal replacement therapy; ECMO, extracorporeal membrane oxygenation; ICU intensive care unit; PTSD, post-traumatic stress disorder.

### Competing risk analysis

Table 3 shows the results of the competing risk analyses, using the Fine and Gray model for suicide and non-suicide mortality after the diagnosis of sepsis. Compared with the 18–35 years group, the 36–50 years group (sHR 2.01, 95% CI 1.46–2.76, *P* < 0.001), 51–65 years group (sHR 2.24, 95% CI 1.64–3.04, *P* < 0.001), 66–80 years group (sHR 2.01, 95% CI 1.46–2.76, *P* < 0.001) and ≥ 81 years group (sHR 3.00, 95% CI 2.21–4.07, *P* < 0.001) were associated with a higher risk of suicide mortality. Male gender (sHR 1.52, 95% CI 1.42–1.66, *P* < 0.001) and living in a rural area (sHR 1.52, 95% CI 1.42–1.66, *P* < 0.001) were associated with a higher risk of suicide mortality. Compared with the quartile 1 group of annual case volume of hospital admissions for sepsis, the quartile 3 (sHR 0.87, 95% CI 0.80–0.95, *P* = 0.002) and quartile 4 (sHR 0.45, 95% CI 0.38–0.52, *P* < 0.001) groups were associated with a lower risk of suicide mortality. Patients with sepsis who underwent CRRT (sHR 1.26, 95% CI 1.07–1.49, *P* = 0.007) and mechanical ventilator support (sHR 2.09, 95% CI 1.90–2.30, *P* < 0.001) had a higher risk of suicide mortality.

**Table 3:** Competing risk analyses using the Fine and Gray model for suicide and non-suicide mortality after the diagnosis of sepsis

Variable	Death by suicide, sHR (95% CI)	*P*-value	Death by other cause, sHR (95% CI)	*P*-value
Age, years				
18–35	1		1	
36–50	2.01 (1.46–2.76)	<0.001	3.38 (3.09–3.69)	<0.001
51–65	2.24 (1.64–3.04)	<0.001	5.47 (5.03–5.95)	<0.001
66–80	2.26 (1.67–3.06)	<0.001	8.06 (7.42–8.86)	<0.001
≥81	3.00 (2.21–4.07)	<0.001	12.22 (11.24–13.28)	<0.001
Gender, male	1.52 (1.42–1.66)	<0.001	1.25 (1.24–1.27)	<0.001
Residence at diagnosis of sepsis				
Urban	1		1	
Rural	1.18 (1.10–1.27)	<0.001	1.04 (1.03–1.05)	<0.001
Income level at diagnosis of sepsis				
Quartile 1 (lowest)	1		1	
Quartile 2	0.96 (0.86–1.08)	0.540	0.99 (0.98–1.02)	0.760
Quartile 3	0.99 (0.89–1.10)	0.830	0.94 (0.92–0.96)	<0.001
Quartile 4 (highest)	0.93 (0.84–1.02)	0.110	0.89 (0.88–0.91)	<0.001
Unknown	0.93 (0.84–1.04)	0.200	1.01 (0.99–1.03)	0.244
Charlson Comorbidity Index				
3–6 (*v*. −2)	1.00 (0.94–1.10)	0.865	1.08 (1.07–1.11)	<0.001
7–9 (*v*. −2)	1.15 (1.06–1.30)	0.002	1.14 (1.11–1.16)	<0.001
−10 (*v*. −2)	1.31 (1.20–1.43)	<0.001	1.34 (1.31–1.37)	<0.001
Elixhauser Comorbidity Index				
8–17 (*v*. −7)	1.31 (1.18–1.44)	<0.001	1.17 (1.15–1.19)	<0.001
18–27 (*v*. −7)	1.49 (1.34–1.66)	<0.001	1.30 (1.27–1.32)	<0.001
−28 (*v*. −7)	1.83 (1.62–2.08)	<0.001	1.34 (1.31–1.37)	<0.001
Admitting department				
Medical department (versus surgical department)	0.54 (0.50–0.58)	<0.001	0.96 (0.94–0.97)	<0.001
Annual case volume of sepsis treatment				
Quartile 1, ≤ 235	1		1	
Quartile 2, 236 to < 710	0.97 (0.90–1.05)	0.460	0.89 (0.88–0.90)	<0.001
Quartile 3, 710 to < 1743	0.87 (0.80–0.95)	0.002	0.76 (0.74–0.77)	<0.001
Quartile 4, ≥ 1743	0.45 (0.38–0.52)	<0.001	0.52 (0.51–0.53)	<0.001
CRRT use	1.26 (1.07–1.49)	0.007	1.49 (1.45–1.53)	<0.001
Vasopressor use	1.06 (0.97–1.16)	0.190	1.68 (1.66–1.71)	<0.001
ECMO support	1.37 (0.71–2.67)	0.350	1.79 (1.57–2.05)	<0.001
Mechanical ventilator support	2.09 (1.90–2.30)	<0.001	3.09 (3.04–3.14)	<0.001
ICU admission	0.92 (0.84–1.01)	0.081	0.87 (0.84–0.87)	<0.001
Total number of hospital admissions for sepsis				
1	1		1	
2–3	0.99 (0.92–1.06)	0.740	0.67 (0.66–0.68)	<0.001
4–5	0.94 (0.82–1.08)	0.380	0.47 (0.45–0.48)	<0.001
6–7	0.80 (0.62–1.02)	0.071	0.36 (0.34–0.37)	<0.001
≥8	0.63 (0.48–0.82)	<0.001	0.21 (0.20–0.22)	<0.001
Concurrent psychiatric illness				
Depression	1.01 (0.94–1.09)	0.760	0.80 (0.79–0.81)	<0.001
Anxiety disorder	0.94 (0.87–1.01)	0.085	0.78 (0.78–0.80)	<0.001
Substance misuse	1.25 (1.05–1.49)	0.012	0.97 (0.95–1.00)	0.043
PTSD	3.52 (1.44–8.58)	0.006	0.64 (0.44–0.93)	0.019
Bipolar disorder	1.15 (1.05–1.26)	0.003	0.87 (0.85–0.88)	<0.001
Schizophrenia or schizophrenic affective disorder	0.98 (0.84–1.13)	0.740	1.01 (0.98–1.04)	0.410
Dementia	1.31 (1.22–1.40)	<0.001	1.67 (1.65–1.69)	<0.001
History of self-harm or suicidal attempt	8.93 (4.90–16.30)	<0.001	0.83 (0.59–1.15)	0.259
Year of diagnosis of sepsis				
2010	1		1	
2011	0.85 (0.74–0.98)	0.024	0.98 (0.96–1.00)	0.104
2012	1.06 (0.93–1.21)	0.410	0.90 (0.88–0.92)	<0.001
2013	1.04 (0.91–1.19)	0.540	0.85 (0.83–0.87)	<0.001
2014	0.96 (0.84–1.10)	0.530	0.81 (0.79–0.83)	<0.001
2015	0.86 (0.75–0.99)	0.033	0.76 (0.74–0.78)	<0.001
2016	0.80 (0.70–0.92)	0.002	0.64 (0.62–0.66)	<0.001
2017	0.78 (0.68–0.90)	0.001	0.59 (0.57–0.60)	<0.001
2018	0.68 (0.59–0.78)	<0.001	0.49 (0.48–0.50)	<0.001

sHR, subdistribution hazard ratio; CRRT, continuous renal replacement therapy; ECMO, extracorporeal membrane oxygenation; ICU, intensive care unit; PTSD, post-traumatic stress disorder.

Among concurrent psychiatric illnesses, substance misuse (sHR 1.25, 95% CI 1.05–1.49, *P* = 0.012), PTSD (sHR 3.52, 95% CI 1.44–8.58, *P* = 0.006), bipolar disorder (sHR 1.15, 95% CI 1.05–1.49, *P* = 0.003) and dementia (sHR 1.31, 95% CI 1.22–1.40, *P* < 0.001) were associated with a higher risk of suicide mortality. Previous self-harm or suicide attempt (sHR 8.93, 95% CI 4.90–16.30, *P* < 0.001) were associated with a higher risk of suicide mortality. The cumulative incidence of suicide mortality according to previous self-harm or suicide attempt, living in a rural area, age, bipolar disorder, dementia, substance misuse and PTSD are presented in Supplementary Figures 1–7, respectively.

### Other analyses

Supplementary Tables 3 and 4 show the results of the competing risk analyses with the Fine and Gray model for suicide mortality in the male and female groups, respectively. Table 4 shows the results of the competing risk analyses with the Fine and Gray model for suicide mortality after excluding patients with sepsis who had concurrent psychiatric disorders or had a history of self-harm or suicidal attempt. In total, 83 860 patients with sepsis were included in this sensitivity analysis, and 1067 (1.3%) died by suicide. Table 5 shows the results of the competing risk analyses with the Fine and Gray model for suicide mortality in patients with sepsis who were admitted to ICUs. In total, 60 165 patients with sepsis were included in this sensitivity analysis, and 1166 (1.9%) died by suicide.

**Table 4:** competing risk analyses using the Fine and Gray model for suicide mortality after excluding patients with sepsis who had concurrent psychiatric disorders or had a history of self-harm or suicide attempt

Variable	Death by suicide, sHR (95% CI)	*P*-value
Gender, male (versus female)	1.62 (1.43–1.83)	<0.001
Age, years		
18–35	1	
36–50	2.89 (1.79–4.67)	<0.001
51–65	3.15 (1.95–5.08)	<0.001
65–80	3.81 (2.37–6.14)	<0.001
≥81	4.94 (3.06–7.97)	<0.001
Residence at diagnosis of sepsis		
Urban	1	
Rural	1.32 (1.15–1.51)	<0.001
Income level at diagnosis of sepsis		
Quartile 1 (lowest)	1	
Quartile 2	0.91 (0.74–1.13)	0.410
Quartile 3	0.97 (0.80–1.18)	0.780
Quartile 4 (highest)	0.87 (0.72–1.13)	0.410
Unknown	0.88 (0.72–1.08)	0.230
Charlson Comorbidity Index		
3–6 (*v*. −2)	0.88 (0.75–1.10)	0.144
7–9 (*v*. −2)	1.09 (1.02–1.11)	0.013
−10 (*v*. −2)	1.27 (1.18–1.39)	<0.001
Elixhauser Comorbidity Index		
8–17 (*v*. −7)	1.54 (1.29–1.83)	<0.001
18–27 (*v*. −7)	2.08 (1.71–2.53)	<0.001
−28 (*v*. −7)	2.90 (2.28–3.70)	<0.001
Admitting department		
Medical department (versus surgical department)	0.43 (0.37–0.49)	<0.001
Total case volume of sepsis treatment		
Quartile 1, ≤ 235	1	
Quartile 2, 236 to < 710	0.88 (0.76–1.03)	0.110
Quartile 3, 710 to < 1743	0.74 (0.63–0.88)	0.001
Quartile 4, ≥ 1743	0.41 (0.32–0.52)	<0.001
CRRT use	1.08 (0.83–1.42)	0.560
Vasopressor use	1.12 (0.95–1.31)	0.180
ECMO support	1.02 (0.38–2.74)	0.980
Mechanical ventilator support	2.44 (2.05–2.90)	<0.001
ICU admission	1.01 (0.86–1.20)	0.870
Total number of hospital admissions for sepsis		
1	1	
2–3	1.11 (0.97–1.28)	0.130
4–5	0.97 (0.72–1.30)	0.830
6–7	0.74 (0.43–1.28)	0.280
≥8	0.62 (0.36–1.05)	0.073
Year of diagnosis of sepsis		
2010	1	
2011	0.76 (0.61–0.95)	0.015
2012	0.96 (0.78–1.19)	0.710
2013	0.82 (0.65–1.03)	0.084
2014	0.61 (0.48–0.79)	<0.001
2015	0.58 (0.45–0.75)	<0.001
2016	0.64 (0.50–0.83)	0.001
2017	0.66 (0.51–0.86)	0.002
2018	0.74 (0.57–0.97)	0.027

sHR, subdistribution hazard ratio; CRRT, continuous renal replacement therapy; ECMO, extracorporeal membrane oxygenation; ICU, intensive care unit.

**Table 5.** competing risk analyses using the Fine and Gray model for suicide mortality in patients with sepsis who were admitted to ICUs

Variable	Death by suicide sHR (95% CI)	*P*-value
Sex, male (versus female)	1.37 (1.22, 1.55)	<0.001
Age, years		
18–35	1	
36–50	0.84 (0.53, 1.34)	0.470
51–65	0.63 (0.41, 0.97)	0.034
65–80	0.61 (0.40, 0.94)	0.024
≥ 81	0.77 (0.50, 1.18)	0.230
Residence at diagnosis of sepsis		
Urban	1	
Rural	1.25 (1.10, 1.42)	0.001
Income level at diagnosis of sepsis		
Quartile 1 (Lowest)	1	
Quartile 2	0.96 (0.78, 1.19)	0.740
Quartile 3	1.04 (0.85, 1.26)	0.720
Quartile 4 (Highest)	1.03 (0.87, 1.23)	0.710
Unknown	0.95 (0.78, 1.16)	0.630
Charlson comorbidity index		
3–6 (*v*. −2)	1.00 (0.85, 1.19)	0.960
7–9 (*v*. −2)	0.98 (0.84, 1.06)	0.059
−10 (*v*. −2)	1.22 (1.05, 1.38)	0.003
Elixhauser comorbidity index		
8–17 (*v*. −7)	1.27 (1.06, 1.52)	0.009
18–27 (*v*. −7)	1.42 (1.18, 1.72)	<0.001
−28 (*v*. −7)	1.86 (1.49, 2.33)	<0.001
Admitting department		
Medical department (versus Surgical department)	0.34 (0.30, 0.39)	<0.001
Total case volume of sepsis treatment		
Quartile 1, ≤ 235	1	
Quartile 2, 236 to < 710	1.22 (1.02, 1.46)	0.028
Quartile 3, 710 to < 1743	1.09 (0.90, 1.30)	0.380
Quartile 4, ≥ 1743	1.01 (0.80, 1.26)	0.960
CRRT use	1.19 (0.99, 1.42)	0.057
Vasopressor use	1.01 (0.89, 1.15)	0.850
ECMO support	0.98 (0.50, 1.91)	0.960
Mechanical ventilator support	1.81 (1.59, 2.05)	<0.001
Total number of hospital admission for sepsis		
1	1	
2–3	0.77 (0.66, 0.89)	<0.001
4–5	0.60 (0.42, 0.88)	0.009
6–7	0.73 (0.40, 1.32)	0.300
≥ 8	0.33 (0.14, 0.79)	0.013
Concurrent psychiatric illness		
Depression	1.01 (0.88, 1.16)	0.840
Anxiety disorder	0.95 (0.84, 1.08)	0.460
Substance abuse	0.97 (0.76, 1.24)	0.810
PTSD	2.04 (0.28, 14.62)	0.480
Bipolar	1.19 (1.00, 1.41)	0.046
Schizophrenia or schizophrenic affective disorder	0.97 (0.76, 1.24)	0.810
Dementia	1.15 (1.01, 1.31)	0.034
History of Self-harm or suicidal attempt	10.34 (3.94, 27.16)	<0.001
Year of diagnosis of sepsis		
2010	1	
2011	0.85 (0.67, 1.08)	0.190
2012	0.97 (0.77, 1.21)	0.780
2013	1.12 (0.90, 1.40)	0.290
2014	0.83 (0.66, 1.04)	0.110
2015	0.82 (0.64, 1.04)	0.110
2016	0.80 (0.62, 1.04)	0.091
2017	0.67 (0.51, 0.89)	0.005
2018	0.56 (0.42, 0.75)	<0.001

ICU, intensive care unit; sHR, subdistribution hazard ratio; CI, confidence interval; CRRT, continuous renal replacement therapy; ECMO, extracorporeal membrane oxygenation; PTSD, post-traumatic stress disorder.

## Discussion

This population-based cohort study showed that 1.5% of patients died by suicide within 1 year after the diagnosis of sepsis in South Korea. Older age, male gender, living in rural areas, comorbid status (higher CCI or ECI score), admission to a hospital with low annual case volumes, invasive treatment (CRRT and mechanical ventilator use), some concurrent psychiatric disorders (substance misuse, PTSD, bipolar disorder and dementia), and history of self-harm or suicide attempt were potential risk factors for suicide among patients with sepsis. Clinically, the knowledge of the factors associated with suicide might allow for earlier intervention to potentially reduce suicide in patients with sepsis. For example, patients with sepsis who have concurrent psychiatric disorders can receive a psychiatric consultation by a psychiatrist, who will prescribe medication for suicide prevention. Moreover, after hospital discharge, the mental health of patients with sepsis who have risk factors for suicide need to be monitored during out-patient visits.

The suicide prevalence in patients with sepsis was 1.5%, which is higher than that reported in a previous study that demonstrated a rate of 0.2% among ICU survivors.^9^ A previous study by Fernando et al focused on all ICU survivors, including many cases of postoperative ICU admission after elective surgeries.^9^ The disease severity might differ between our study population with sepsis and overall ICU survivors evaluated in the study by Fernando et al.^9^ The patients with sepsis in our study may have had more severe conditions because we included all patients with sepsis, regardless of whether they died during their ICU stay or hospital admission. Meanwhile, the study by Fernando et al included ICU survivors.^9^ The disease severity during hospital admission might explain the discrepancy in the suicide rates between the two studies.

Various concurrent psychiatric disorders were evaluated as potential risk factors for suicide mortality among patients with sepsis. Among the disorders, substance misuse, PTSD, bipolar disorder and dementia were potential risk factors for suicide, whereas depression, anxiety disorder and schizophrenia were not. Interestingly, approximately 90% (10 194/11 467) of substance misuse was alcohol misuse among patients with sepsis in this study. Alcohol misuse was a powerful risk factor for suicide, and the relative risk of suicide in patients who misused alcohol was reported as 6.9% in a previous study.^19^ PTSD is also an important risk factor for suicide,^20^ and patients with PTSD following sepsis may be a high-risk population for suicide. Interestingly, PTSD was a significant risk factor for suicide in only female patients (sHR 4.68), and it was not significant in male patients with sepsis in this study. A recent cohort study in Sweden reported that PTSD accounts for 0.6% of suicides in men and 3.5% in women, suggesting that PTSD is a significant risk factor for suicide, particularly in women.^20^ However, information on the impact of female gender on suicide risk among patients with sepsis is still lacking, and more research on this topic is needed.

Bipolar disorder was also identified as a significant risk factor for suicide in patients with sepsis, regardless of gender, in this study. Bipolar disorder is associated with the highest rate of suicide of all psychiatric disorders, approximately 20–30 times that of the general population.^21^ Fernando et al also reported that concurrent bipolar disorder was a significant risk factor for suicide among ICU survivors.^9^ However, an important psychiatric disorder, depression, was not a risk factor for suicide in our study among patients with sepsis. This is the most significant difference between the findings of Fernando et al and our findings.^9^ Several factors might affect this finding. The median age of our study population was 77 years (IQR 64–84 years), suggesting that many of the patients with sepsis were elderly patients. Depression in older people can be underdiagnosed and undertreated.^22^ It is possible that there may have been missed cases of depression in our study because we used a national registration database according to ICD-10 codes. However, in the subgroup analysis, depression was a significant risk factor for suicide in male patients, but was not significant in female patients. A recent study reported that depression in males was significantly associated with suicide risk independent of conventional depression symptoms.^23^ Moreover, depression symptoms with the greatest risk in men were emotional suppression, substance misuse, somatic symptoms and risk-taking behaviours. Considering that this is the first study to report the relationship between concurrent depression and suicide in patients with sepsis, further study is needed. Specifically, the association between the timing and occurrence of comorbid psychiatric diseases in patients with sepsis and suicide risk should be clarified in a future study.

Dementia was also a powerful risk factor for suicide and was significant, regardless of gender, ICU admission status or study period (2016–2018). The increased risk of suicide among patients with dementia and its prevention are important public health issues.^24^ Moreover, a recent Swedish cohort study reported that dementia was more common in individuals diagnosed with sepsis,^25^ suggesting that prevention of suicide is clinically important in patients with sepsis and dementia. Previous suicide attempt was an important risk factor for death by suicide,^26^ and this also applied in our results. In agreement with the findings by Fernando et al,^9^ we suggest that patients with sepsis with a history of suicide attempt are a high-risk population for suicide.

Old age and male gender were potential risk factors for suicide in patients with sepsis in this study. A recent study reported that old age and male gender were risk factors for suicide among Chinese adults,^27^ and suicide in elderly people is an important public health issue in many countries.^28^ Moreover, urban–rural inequalities in suicide mortality, such as increased risk of suicide in individuals living in rural areas, have also been reported in previous studies.^29–31^ For individuals living in rural areas, social isolation, which results in less intimate face-to-face contact with family and friends, poorer access to mental healthcare facilities and easier access to lethal means, might increase the risk of suicide.^32^

Fernando et al^9^ also reported that ICU survivors who experienced invasive treatment, such as mechanical ventilatory support or CRRT, were at a higher risk of suicide. We also found that invasive treatment (CRRT and mechanical ventilator use) was a risk factor for suicide in patients with sepsis. The use of invasive treatments such as CRRT and mechanical ventilation meant that patients experienced major organ failure owing to sepsis.^33^ Sepsis survivors who developed major organ failure might have post-sepsis syndrome, resulting in a newly acquired disability,^7^ which might be responsible for the increased risk of suicide in our study.

This study has several limitations. First, we did not consider the severity of sepsis by using accurate tools such as the Simplified Acute Physiology Score II or Acute Physiology and Chronic Health Evaluation II. As the severity of sepsis affects the risk of suicide in patients with sepsis, this might have affected our results. Second, some information pertaining to factors such as body mass index, marital status, history of smoking and alcohol consumption was not included in this study because they were unavailable in the NHIS database. Third, we used the ICD-10 codes for sepsis to extract the data of the study population from the national registration database in South Korea. However, some cases may have been missed by this method because some patients with sepsis were not registered as sepsis cases based on ICD-10 codes in the NHIS database. Fourth, considering the old age and gender imbalance, it is difficult to conclude that our study population was a representative sample of the common population of patients with sepsis in South Korea. For example, elderly people have a high risk of depression because of loneliness and loss of significant others;^34^ this affected the prevalence of suicide in this study. Moreover, patients with chronic conditions, such as diabetes mellitus, cancer and other diseases, are more prone to sepsis and might have a lower quality of life before the diagnosis of sepsis. Finally, we used the ICD-10 codes for calculating the CCI and ECI score; however, the actual underlying diseases of patients with sepsis might differ. For example, some patients were not diagnosed in the NHIS database because of mild symptoms or poor access to healthcare resources.

In conclusion, during the 1-year follow-up period, 1.5% of patients with a diagnosis of sepsis died by suicide in South Korea. Factors such as old age, male gender, living in rural areas, comorbid status, admission to a hospital with low annual case volumes, invasive treatment, concurrent PTSD, bipolar disorder, substance misuse, dementia and history of self-harm or suicide attempt were potential risk factors for suicide among patients with sepsis. The knowledge of factors associated with suicide could allow for earlier intervention to potentially reduce the number of suicide attempts in patients with sepsis. Future research should identify methods of reducing the number of suicide attempts in patients with sepsis, particularly those with these additional prognostic factors.

## Data Availability

Data are available upon reasonable request. Anonymised data used in the present study may be available upon reasonable request to the corresponding author, I.-A.S.

## Supplementary material

For supplementary material accompanying this paper visit http://doi.org/10.1192/bjo.2022.19. click here to view supplementary material
