# Supplementary material for: Prevalence and risk factors for suicide in patients with sepsis: nationwide cohort study in South Korea
Source: BJPsych Open. 2022 Mar 10;8(2):e61. doi: 10.1192/bjo.2022.19 (PMC8935909; doi:10.1192/bjo.2022.19)

**Table S1. ICD-10 codes**

The ICD-10 codes used by comorbidity to compute the Charlson comorbidity index are:

- Myocardial infarction: I21.x, I22.x, I25.2
- Congestive heart failure: I09.9, I11.0, I13.0, I13.2, I25.5, I42.0, I42.5 - I42.9, I43.x, I50.x, P29.0
- Peripheral vascular disease: I70.x, I71.x, I73.1, I73.8, I73.9, I77.1, I79.0, I79.2, K55.1, K55.8, K55.9, Z95.8, Z95.9
- Cerebrovascular disease: G45.x, G46.x, H34.0, I60.x - I69.x
- Dementia: F00.x - F03.x, F05.1, G30.x, G31.1
- Chronic pulmonary disease: I27.8, I27.9, J40.x - J47.x, J60.x - J67.x, J68.4, J70.1, J70.3
- Rheumatic disease: M05.x, M06.x, M31.5, M32.x - M34.x, M35.1, M35.3, M36.0
- Peptic ulcer disease: K25.x - K28.x
- Mild liver disease: B18.x, K70.0 - K70.3, K70.9, K71.3 - K71.5, K71.7, K73.x, K74.x, K76.0, K76.2 - K76.4, K76.8, K76.9, Z94.4
- Diabetes without chronic complication: E10.0, E10.1, E10.6, E10.8, E10.9, E11.0, E11.1, E11.6, E11.8, E11.9, E12.0, E12.1, E12.6, E12.8, E12.9, E13.0, E13.1, E13.6, E13.8, E13.9, E14.0, E14.1, E14.6, E14.8, E14.9
- Diabetes with chronic complication: E10.2 - E10.5, E10.7, E11.2 - E11.5, E11.7, E12.2 - E12.5, E12.7, E13.2 - E13.5, E13.7, E14.2 - E14.5, E14.7
- Hemiplegia or paraplegia: G04.1, G11.4, G80.1, G80.2, G81.x, G82.x, G83.0 - G83.4, G83.9
- Renal disease: I12.0, I13.1, N03.2 - N03.7, N05.2 - N05.7, N18.x, N19.x, N25.0, Z49.0 - Z49.2, Z94.0, Z99.2
- Any malignancy, including lymphoma and leukaemia, except malignant neoplasm of skin: C00.x - C26.x, C30.x - C34.x, C37.x - C41.x, C43.x, C45.x - C58.x, C60.x - C76.x, C81.x - C85.x, C88.x, C90.x - C97.x
- Moderate or severe liver disease: I85.0, I85.9, I86.4, I98.2, K70.4, K71.1, K72.1, K72.9, K76.5, K76.6, K76.7
- Metastatic solid tumour: C77.x - C80.x
- AIDS/HIV: B20.x - B22.x, B24.x

**Table S2. ICD-10 codes**

The ICD-10 codes used by comorbidity to compute the Elixhauser comorbidity index are:

- Congestive heart failure: I09.9, I11.0, I13.0, I13.2, I25.5, I42.0, I42.5 - I42.9, I43.x, I50.x, P29.0
- Cardiac arrhythmias: I44.1 - I44.3, I45.6, I45.9, I47.x - I49.x, R00.0, R00.1, R00.8, T82.1, Z45.0, Z95.0
- Valvular disease: A52.0, I05.x - I08.x, I09.1, I09.8, I34.x - I39.x, Q23.0 - Q23.3, Z95.2 - Z95.4
- Pulmonary circulation disorders: I26.x, I27.x, I28.0, I28.8, I28.9
- Peripheral vascular disorders: I70.x, I71.x, I73.1, I73.8, I73.9, I77.1, I79.0, I79.2, K55.1, K55.8, K55.9, Z95.8, Z95.9
- Hypertension, uncomplicated: I10.x
- Hypertension, complicated: I11.x - I13.x, I15.x
- Paralysis: G04.1, G11.4, G80.1, G80.2, G81.x, G82.x, G83.0 - G83.4, G83.9
- Other neurological disorders: G10.x - G13.x, G20.x - G22.x, G25.4, G25.5, G31.2, G31.8, G31.9, G32.x, G35.x - G37.x, G40.x, G41.x, G93.1, G93.4, R47.0, R56.x
- Chronic pulmonary disease: I27.8, I27.9, J40.x - J47.x, J60.x - J67.x, J68.4, J70.1, J70.3
- Diabetes, uncomplicated: E10.0, E10.1, E10.9, E11.0, E11.1, E11.9, E12.0, E12.1, E12.9, E13.0, E13.1, E13.9, E14.0, E14.1, E14.9
- Diabetes, complicated: E10.2 - E10.8, E11.2 - E11.8, E12.2 - E12.8, E13.2 - E13.8, E14.2 - E14.8
- Hypothyroidism: E00.x - E03.x, E89.0
- Renal failure: I12.0, I13.1, N18.x, N19.x, N25.0, Z49.0 - Z49.2, Z94.0, Z99.2
- Liver disease: B18.x, I85.x, I86.4, I98.2, K70.x, K71.1, K71.3 - K71.5, K71.7, K72.x - K74.x, K76.0, K76.2 - K76.9, Z94.4
- Peptic ulcer disease, excluding bleeding: K25.7, K25.9, K26.7, K26.9, K27.7, K27.9, K28.7, K28.9
- AIDS/HIV: B20.x - B22.x, B24.x
- Lymphoma: C81.x - C85.x, C88.x, C96.x, C90.0, C90.2
- Metastatic cancer: C77.x - C80.x
- Solid tumour without metastasis: C00.x - C26.x, C30.x - C34.x, C37.x - C41.x, C43.x, C45.x - C58.x, C60.x - C76.x, C97.x
- Rheumatoid arthritis/collagen vascular diseases: L94.0, L94.1, L94.3, M05.x, M06.x, M08.x, M12.0, M12.3, M30.x, M31.0 - M31.3, M32.x - M35.x, M45.x, M46.1, M46.8, M46.9
- Coagulopathy: D65 - D68.x, D69.1, D69.3 - D69.6
- Obesity: E66.x
- Weight loss: E40.x - E46.x, R63.4, R64
- Fluid and electrolyte disorders: E22.2, E86.x, E87.x
- Blood loss anaemia: D50.0
- Deficiency anaemia: D50.8, D50.9, D51.x - D53.x
- Alcohol abuse: F10, E52, G62.1, I42.6, K29.2, K70.0, K70.3, K70.9, T51.x, Z50.2, Z71.4, Z72.1
- Drug abuse: F11.x - F16.x, F18.x, F19.x, Z71.5, Z72.2
- Psychoses: F20.x, F22.x - F25.x, F28.x, F29.x, F30.2, F31.2, F31.5
- Depression: F20.4, F31.3 - F31.5, F32.x, F33.x, F34.1, F41.2, F43.2

Table S3. Competing risk analyses using the Fine and Gray model for suicide mortality in the male

| Variable | | Death by suicide  sHR (95% CI) | *P*-value |
| --- | --- | --- | --- |
| Age, year | |  |  |
|  | 18-35 | 1 |  |
|  | 36-50 | 1·99 (1·32, 3·00) | 0·001 |
|  | 51-65 | 2·31 (1·55, 3·44) | <0·001 |
|  | 65-80 | 2·19 (1·47, 3·26) | <0·001 |
|  | ≥ 81 | 2·24 (1·50, 3·35) | <0·001 |
| Residence at diagnosis of sepsis | |  |  |
|  | Urban | 1 |  |
|  | Rural | 1·26 (1·14, 1·39) | <0·001 |
| Income level at diagnosis of sepsis | |  |  |
|  | Q1 (Lowest) | 1 |  |
|  | Q2 | 0·94 (0·81, 1·10) | 0·450 |
|  | Q3 | 0·97 (0·84, 1·12) | 0·700 |
|  | Q4 (Highest) | 0·89 (0·78, 1·02) | 0·085 |
|  | Unknown | 0·81 (0·69, 0·94) | 0·006 |
| Charlson comorbidity index | |  |  |
|  | 3-6 (vs -2) | 1·00 (0·94, 1·09 | 0·720 |
|  | 7-9 (vs -2) | 1·14(1·05 1·30) | 0·001 |
|  | -10 (vs -2) | 1·30 (1·21, 1·45) | <0·001 |
| Elixhauser comorbidity index | |  |  |
|  | 8-17 (vs -7) | 1·47 (1·27, 1·70) | <0·001 |
|  | 18-27 (vs -7) | 1·85 (1·59, 2·16) | <0·001 |
|  | -28 (vs -7) | 2·37 (1·99, 2·83) | <0·001 |
| Admitting department | |  |  |
|  | Medical department (vs Surgical department) | 0·52 (0·46, 0·58) | <0·001 |
| Total case volume of sepsis treatment | |  |  |
|  | Q1 ≤ 235 | 1 |  |
|  | 236 ≤ Q2 ≤ 710 | 0·95 (0·86, 1·06) | 0·360 |
|  | 710 ≤ Q3 ≤ 1743 | 0·83 (0·74, 0·94) | 0·003 |
|  | Q4 ≥ 1743 | 0·42 (0·34, 0·51) | <0·001 |
| CRRT use | | 1·16 (0·94, 1·44) | 0·170 |
| Vasopressor use | | 1·13 (1·00, 1·28) | 0·043 |
| ECMO support | | 1·12 (0·50, 2·52) | 0·790 |
| Mechanical ventilator support | | 2·02 (1·77, 2·30) | <0·001 |
| ICU admission | | 0·90 (0·79, 1·02) | 0·097 |
| Total number of hospital admission for sepsis | |  |  |
|  | 1 | 1 |  |
|  | 2-3 | 1·08 (0·98, 1·19) | 0·110 |
|  | 4-5 | 1·19 (1·00, 1·42) | 0·052 |
|  | 6-7 | 0·90 (0·65, 1·25) | 0·540 |
|  | ≥ 8 | 0·86 (0·63, 1·16) | 0·320 |
| Concurrent psychiatric illness | |  |  |
|  | Depression | 1·11 (1·00, 1·23) | 0·048 |
|  | Anxiety disorder | 0·92 (0·84, 1·02) | 0·130 |
|  | Substance abuse | 1·05 (0·87, 1·28) | 0·590 |
|  | PTSD | 2·82 (0·86, 9·18) | 0·086 |
|  | Bipolar | 1·16 (1·02, 1·31) | 0·025 |
|  | Schizophrenia or schizophrenic affective disorder | 0·90 (0·74, 1·10) | 0·310 |
|  | Dementia | 1·16 (1·05, 1·28) | 0·004 |
| History of Self-harm or suicidal attempt | | 8·28 (3·95, 17·36) | <0·001 |
| Year of diagnosis of sepsis | |  |  |
|  | 2010 | 1 |  |
|  | 2011 | 0·79 (0·66, 0·95) | 0·014 |
|  | 2012 | 1·01 (0·85, 1·21) | 0·870 |
|  | 2013 | 0·86 (0·72, 1·03) | 0·110 |
|  | 2014 | 0·82 (0·69, 0·99) | 0·036 |
|  | 2015 | 0·77 (0·64, 0·93) | 0·006 |
|  | 2016 | 0·72 (0·59, 0·87) | 0·001 |
|  | 2017 | 0·71 (0·59, 0·86) | <0·001 |
|  | 2018 | 0·66 (0·55, 0·81) | <0·001 |

sHR, subdistribution hazard ratio; CI, confidence interval; CRRT, continuous renal replacement therapy; ECMO, extracorporeal membrane oxygenation; ICU, intensive care unit; PTSD, post-traumatic stress disorder

Table S4. Competing risk analyses using the Fine and Gray model for suicide mortality in the female

| Variable | | Death by suicide  sHR (95% CI) | *P*-value |
| --- | --- | --- | --- |
| Age, year | |  |  |
|  | 18-35 | 1 |  |
|  | 36-50 | 1·85 (1·10, 3·11) | 0·020 |
|  | 51-65 | 1·55 (0·94, 2·56) | 0·088 |
|  | 65-80 | 2·36 (1·47, 3·80) | <0·001 |
|  | ≥ 81 | 3·93 (2·45, 6·31) | <0·001 |
| Residence at diagnosis of sepsis | |  |  |
|  | Urban | 1 |  |
|  | Rural | 1·11 (1·00, 1·23) | 0·047 |
| Income level at diagnosis of sepsis | |  |  |
|  | Q1 (Lowest) | 1 |  |
|  | Q2 | 1·00 (0·84, 1·19) | 0·990 |
|  | Q3 | 1·04 (0·89, 1·21) | 0·650 |
|  | Q4 (Highest) | 1·04 (0·91, 1·18) | 0·610 |
|  | Unknown | 1·03 (0·89, 1·20) | 0·680 |
| Charlson comorbidity index | |  |  |
|  | 3-6 (vs -2) | 0·86 (0·75, 1·12) | 0·213 |
|  | 7-9 (vs -2) | 1·12 (1·02 1·21) | 0·013 |
|  | -10 (vs -2) | 1·34 (1·20, 1·47) | <0·001 |
| Elixhauser comorbidity index | |  |  |
|  | 8-17 (vs -7) | 1·18 (1·10, 3·11) | 0·020 |
|  | 18-27 (vs -7) | 1·19 (1·02, 1·38) | 0·030 |
|  | -28 (vs -7) | 1·38 (1·15, 1·65) | 0·001 |
| Admitting department | |  |  |
|  | Medical department (vs Surgical department) | 0·57 (0·51, 0·65) | <0·001 |
| Total case volume of sepsis treatment | |  |  |
|  | Q1 ≤ 235 | 1 |  |
|  | 236 ≤ Q2 ≤ 710 | 1·00 (0·89, 1·11) | 0·930 |
|  | 710 ≤ Q3 ≤ 1743 | 0·93 (0·82, 1·06) | 0·280 |
|  | Q4 ≥ 1743 | 0·53 (0·42, 0·67) | <0·001 |
| CRRT use | | 1·39 (1·06, 1·83) | 0·017 |
| Vasopressor use | | 0·97 (0·84, 1·11) | 0·620 |
| ECMO support | | 2·30 (0·73, 7·18) | 0·150 |
| Mechanical ventilator support | | 2·21 (1·92, 2·54) | <0·001 |
| ICU admission | | 0·97 (0·85, 1·10) | 0·630 |
| Total number of hospital admission for sepsis | |  |  |
|  | 1 | 1 |  |
|  | 2-3 | 0·88 (0·80, 0·98) | 0·021 |
|  | 4-5 | 0·69 (0·55, 0·86) | 0·001 |
|  | 6-7 | 0·68 (0·47, 0·99) | 0·047 |
|  | ≥ 8 | 0·36 (0·22, 0·59) | <0·001 |
| Concurrent psychiatric illness | |  |  |
|  | Depression | 0·93 (0·84, 1·04) | 0·220 |
|  | Anxiety disorder | 0·98 (0·88, 1·08) | 0·650 |
|  | Substance abuse | 1·94 (1·26, 2·97) | 0·002 |
|  | PTSD | 4·68 (1·13, 19·38) | 0·033 |
|  | Bipolar | 1·16 (1·01, 1·33) | 0·034 |
|  | Schizophrenia or schizophrenic affective disorder | 1·08 (0·87, 1·34) | 0·480 |
|  | Dementia | 1·50 (1·36, 1·66) | <0·001 |
| History of Self-harm or suicidal attempt | | 9·45 (3·32, 26·91) | <0·001 |
| Year of diagnosis of sepsis | |  |  |
|  | 2010 | 1 |  |
|  | 2011 | 0·90 (0·73, 1·11) | 0·330 |
|  | 2012 | 1·07 (0·88, 1·31) | 0·480 |
|  | 2013 | 1·24 (1·02, 1·51) | 0·031 |
|  | 2014 | 1·09 (0·89, 1·33) | 0·410 |
|  | 2015 | 0·94 (0·76, 1·16) | 0·560 |
|  | 2016 | 0·87 (0·70, 1·07) | 0·180 |
|  | 2017 | 0·84 (0·68, 1·03) | 0·095 |
|  | 2018 | 0·65 (0·52, 0·81) | <0·001 |

sHR, subdistribution hazard ratio; CI, confidence interval; CRRT, continuous renal replacement therapy; ECMO, extracorporeal membrane oxygenation; ICU, intensive care unit; PTSD, post-traumatic stress disorder

Table S5. Competing risk analyses using the Fine and Gray model for suicide mortality in patients with sepsis who were admitted to hospitals from 2016 to 2018

| Variable | | Death by suicide  sHR (95% CI) | *P*-value |
| --- | --- | --- | --- |
| Sex, male (vs female) | | 1·62 (1·45, 1·82) | <0·001 |
| Age, year | |  |  |
|  | 18-35 | 1 |  |
|  | 36-50 | 2·00 (1·06, 3·78) | 0·032 |
|  | 51-65 | 2·25 (1·23, 4·13) | 0·009 |
|  | 65-80 | 2·43 (1·33, 4·44) | 0·004 |
|  | ≥ 81 | 3·42 (1·87, 6·25) | <0·001 |
| Residence at diagnosis of sepsis | |  |  |
|  | Urban | 1 |  |
|  | Rural | 1·04 (0·92, 1·17) | 0·520 |
| Income level at diagnosis of sepsis | |  |  |
|  | Q1 (Lowest) | 1 |  |
|  | Q2 | 1·07 (0·88, 1·30) | 0·510 |
|  | Q3 | 1·18 (0·99, 1·41) | 0·060 |
|  | Q4 (Highest) | 1·10 (0·94, 1·28) | 0·240 |
|  | Unknown | 1·13 (0·92, 1·39) | 0·240 |
| Charlson comorbidity index | |  |  |
|  | 3-6 (vs -2) | 1·02 (0·87, 1·21) | 0·960 |
|  | 7-9 (vs -2) | 1·05 (0·84, 1·06) | 0·062 |
|  | -10 (vs -2) | 1·35 (1·15, 1·48) | <0·001 |
| Elixhauser comorbidity index | |  |  |
|  | 8-17 (vs -7) | 1·47 (1·18, 1·83) | 0·002 |
|  | 18-27 (vs -7) | 1·61 (1·28, 2·02) | <0·001 |
|  | -28 (vs -7) | 1·87 (1·47, 2·38) | <0·001 |
| Admitting department | |  |  |
|  | Medical department (vs Surgical department) | 1·04 (0·92, 1·17) | 0·520 |
| Total case volume of sepsis treatment | |  |  |
|  | Q1 ≤ 235 | 1 |  |
|  | 236 ≤ Q2 ≤ 710 | 0·92 (0·81, 1·05) | 0·210 |
|  | 710 ≤ Q3 ≤ 1743 | 0·89 (0·76, 1·04) | 0·140 |
|  | Q4 ≥ 1743 | 0·53 (0·41, 0·69) | <0·001 |
| CRRT use | | 1·11 (0·80, 1·53) | 0·540 |
| Vasopressor use | | 1·16 (0·99, 1·36) | 0·062 |
| ECMO support | | 1·79 (0·57, 5·67) | 0·320 |
| Mechanical ventilator support | | 2·31 (1·90, 2·81) | <0·001 |
| ICU admission | | 0·83 (0·69, 1·00) | 0·045 |
| Total number of hospital admission for sepsis | |  |  |
|  | 1 | 1 |  |
|  | 2-3 | 1·08 (0·96, 1·23) | 0·220 |
|  | 4-5 | 1·05 (0·83, 1·32) | 0·680 |
|  | 6-7 | 0·92 (0·61, 1·40) | 0·700 |
|  | ≥ 8 | 1·35 (0·96, 1·91) | 0·087 |
| Concurrent psychiatric illness | |  |  |
|  | Depression | 0·88 (0·76, 1·01) | 0·053 |
|  | Anxiety disorder | 0·99 (0·87, 1·12) | 0·810 |
|  | Substance abuse | 1·48 (1·20, 1·83) | <0·001 |
|  | PTSD | 3·54 (0·89, 14·10) | 0·073 |
|  | Bipolar | 1·20 (1·05, 1·37) | 0·008 |
|  | Schizophrenia or schizophrenic affective disorder | 1·00 (0·79, 1·28) | 0·970 |
|  | Dementia | 1·29 (1·14, 1·47) | <0·001 |
| History of Self-harm or suicidal attempt | | 9·68 (3·49, 26·89) | <0·001 |
| Year of diagnosis of sepsis | |  |  |
|  | 2016 | 1 |  |
|  | 2017 | 0·97 (0·85, 1·11) | 0·660 |
|  | 2018 | 0·88 (0·76, 1·02) | 0·088 |

sHR, subdistribution hazard ratio; CI, confidence interval; CRRT, continuous renal replacement therapy; ECMO, extracorporeal membrane oxygenation; ICU, intensive care unit; PTSD, post-traumatic stress disorder

Figure S1.


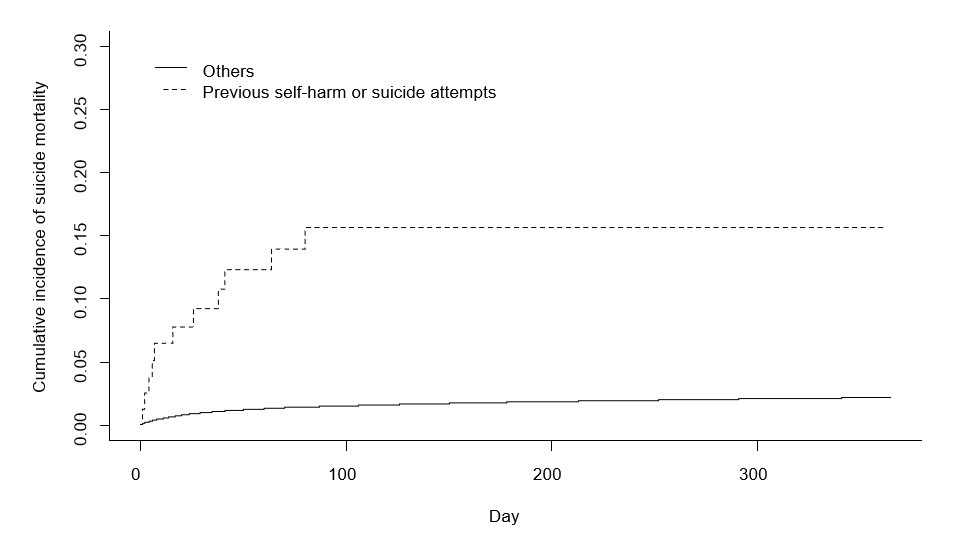


Figure S2


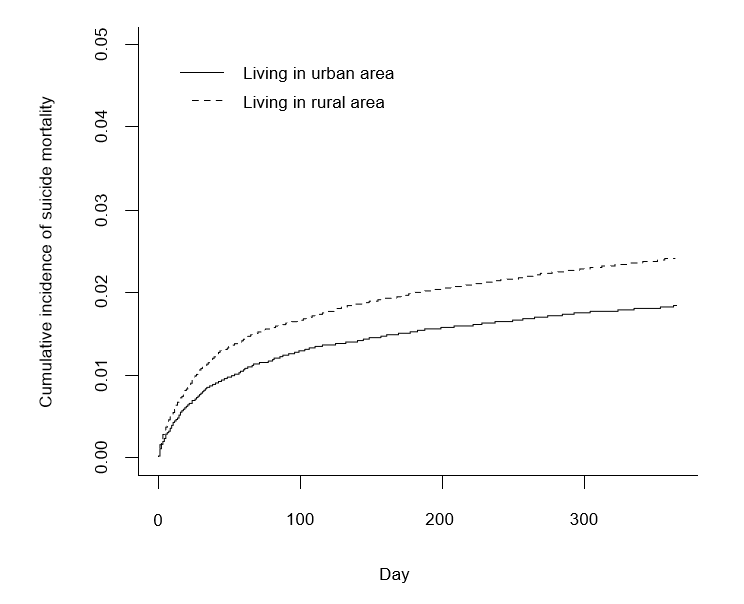


Figure S3.


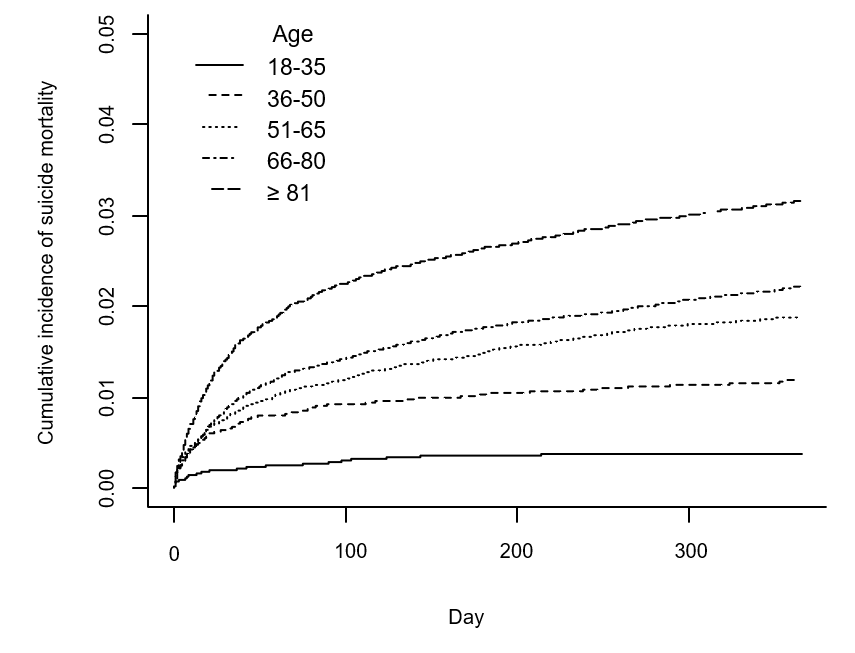


Figure S4.


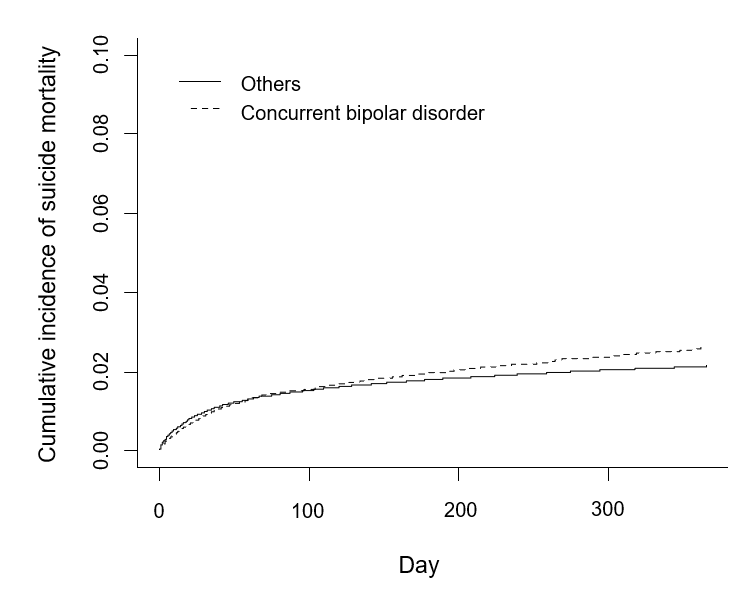


Figure S5.


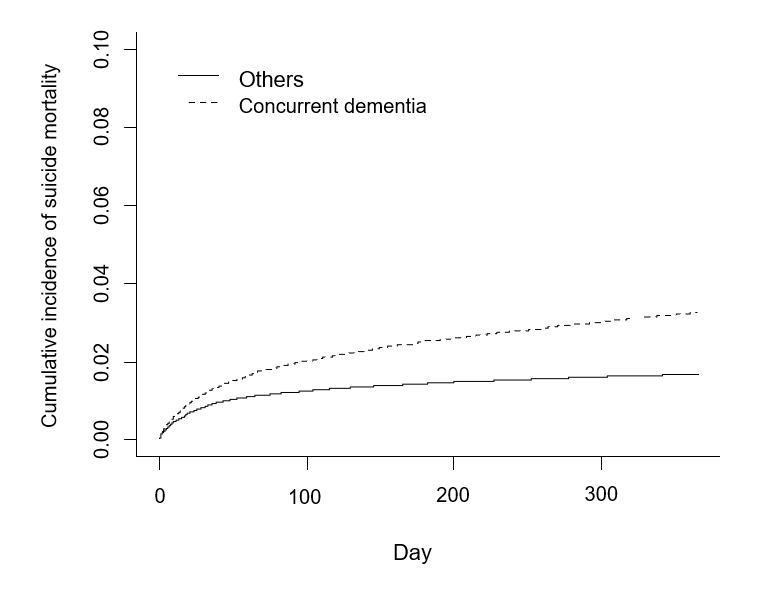


Figure S6.


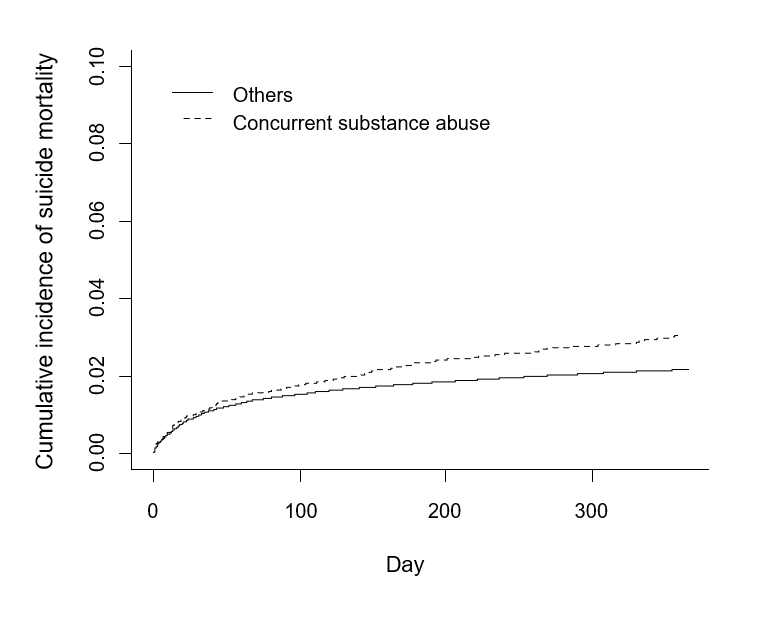


Figure S7.


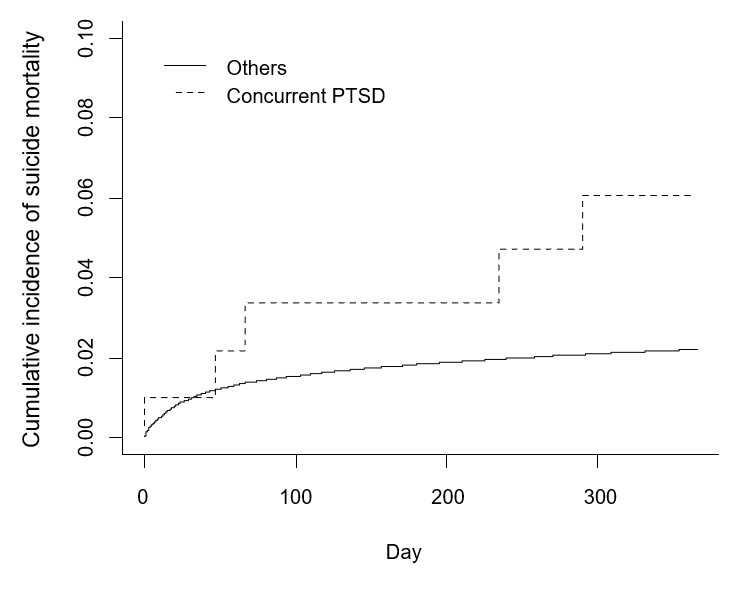

Supplement: Supplementary file 1 [file S2056472422000199sup001.zip › S2056472422000199sup008.docx]
