# Supplementary material for: Prevalence and risk factors for suicide in patients with sepsis: nationwide cohort study in South Korea
Source: BJPsych Open. 2022 Mar 10;8(2):e61. doi: 10.1192/bjo.2022.19 (PMC8935909; doi:10.1192/bjo.2022.19)
Supplement: Supplementary file 1 [file S2056472422000199sup001.zip › S2056472422000199sup009.docx]

**Table S2. ICD-10 codes**

The ICD-10 codes used by comorbidity to compute the Elixhauser comorbidity index are:

- Congestive heart failure: I09.9, I11.0, I13.0, I13.2, I25.5, I42.0, I42.5 - I42.9, I43.x, I50.x, P29.0
- Cardiac arrhythmias: I44.1 - I44.3, I45.6, I45.9, I47.x - I49.x, R00.0, R00.1, R00.8, T82.1, Z45.0, Z95.0
- Valvular disease: A52.0, I05.x - I08.x, I09.1, I09.8, I34.x - I39.x, Q23.0 - Q23.3, Z95.2 - Z95.4
- Pulmonary circulation disorders: I26.x, I27.x, I28.0, I28.8, I28.9
- Peripheral vascular disorders: I70.x, I71.x, I73.1, I73.8, I73.9, I77.1, I79.0, I79.2, K55.1, K55.8, K55.9, Z95.8, Z95.9
- Hypertension, uncomplicated: I10.x
- Hypertension, complicated: I11.x - I13.x, I15.x
- Paralysis: G04.1, G11.4, G80.1, G80.2, G81.x, G82.x, G83.0 - G83.4, G83.9
- Other neurological disorders: G10.x - G13.x, G20.x - G22.x, G25.4, G25.5, G31.2, G31.8, G31.9, G32.x, G35.x - G37.x, G40.x, G41.x, G93.1, G93.4, R47.0, R56.x
- Chronic pulmonary disease: I27.8, I27.9, J40.x - J47.x, J60.x - J67.x, J68.4, J70.1, J70.3
- Diabetes, uncomplicated: E10.0, E10.1, E10.9, E11.0, E11.1, E11.9, E12.0, E12.1, E12.9, E13.0, E13.1, E13.9, E14.0, E14.1, E14.9
- Diabetes, complicated: E10.2 - E10.8, E11.2 - E11.8, E12.2 - E12.8, E13.2 - E13.8, E14.2 - E14.8
- Hypothyroidism: E00.x - E03.x, E89.0
- Renal failure: I12.0, I13.1, N18.x, N19.x, N25.0, Z49.0 - Z49.2, Z94.0, Z99.2
- Liver disease: B18.x, I85.x, I86.4, I98.2, K70.x, K71.1, K71.3 - K71.5, K71.7, K72.x - K74.x, K76.0, K76.2 - K76.9, Z94.4
- Peptic ulcer disease, excluding bleeding: K25.7, K25.9, K26.7, K26.9, K27.7, K27.9, K28.7, K28.9
- AIDS/HIV: B20.x - B22.x, B24.x
- Lymphoma: C81.x - C85.x, C88.x, C96.x, C90.0, C90.2
- Metastatic cancer: C77.x - C80.x
- Solid tumour without metastasis: C00.x - C26.x, C30.x - C34.x, C37.x - C41.x, C43.x, C45.x - C58.x, C60.x - C76.x, C97.x
- Rheumatoid arthritis/collagen vascular diseases: L94.0, L94.1, L94.3, M05.x, M06.x, M08.x, M12.0, M12.3, M30.x, M31.0 - M31.3, M32.x - M35.x, M45.x, M46.1, M46.8, M46.9
- Coagulopathy: D65 - D68.x, D69.1, D69.3 - D69.6
- Obesity: E66.x
- Weight loss: E40.x - E46.x, R63.4, R64
- Fluid and electrolyte disorders: E22.2, E86.x, E87.x
- Blood loss anaemia: D50.0
- Deficiency anaemia: D50.8, D50.9, D51.x - D53.x
- Alcohol abuse: F10, E52, G62.1, I42.6, K29.2, K70.0, K70.3, K70.9, T51.x, Z50.2, Z71.4, Z72.1
- Drug abuse: F11.x - F16.x, F18.x, F19.x, Z71.5, Z72.2
- Psychoses: F20.x, F22.x - F25.x, F28.x, F29.x, F30.2, F31.2, F31.5
- Depression: F20.4, F31.3 - F31.5, F32.x, F33.x, F34.1, F41.2, F43.2
