# Supplementary material for: Prevalence and risk factors for suicide in patients with sepsis: nationwide cohort study in South Korea
Source: BJPsych Open. 2022 Mar 10;8(2):e61. doi: 10.1192/bjo.2022.19 (PMC8935909; doi:10.1192/bjo.2022.19)
Supplement: Supplementary file 1 [file S2056472422000199sup001.zip › S2056472422000199sup010.docx]

Table S3. Competing risk analyses using the Fine and Gray model for suicide mortality in the male

| Variable | | Death by suicide  sHR (95% CI) | *P*-value |
| --- | --- | --- | --- |
| Age, year | |  |  |
|  | 18-35 | 1 |  |
|  | 36-50 | 1·99 (1·32, 3·00) | 0·001 |
|  | 51-65 | 2·31 (1·55, 3·44) | <0·001 |
|  | 65-80 | 2·19 (1·47, 3·26) | <0·001 |
|  | ≥ 81 | 2·24 (1·50, 3·35) | <0·001 |
| Residence at diagnosis of sepsis | |  |  |
|  | Urban | 1 |  |
|  | Rural | 1·26 (1·14, 1·39) | <0·001 |
| Income level at diagnosis of sepsis | |  |  |
|  | Q1 (Lowest) | 1 |  |
|  | Q2 | 0·94 (0·81, 1·10) | 0·450 |
|  | Q3 | 0·97 (0·84, 1·12) | 0·700 |
|  | Q4 (Highest) | 0·89 (0·78, 1·02) | 0·085 |
|  | Unknown | 0·81 (0·69, 0·94) | 0·006 |
| Charlson comorbidity index | |  |  |
|  | 3-6 (vs -2) | 1·00 (0·94, 1·09 | 0·720 |
|  | 7-9 (vs -2) | 1·14(1·05 1·30) | 0·001 |
|  | -10 (vs -2) | 1·30 (1·21, 1·45) | <0·001 |
| Elixhauser comorbidity index | |  |  |
|  | 8-17 (vs -7) | 1·47 (1·27, 1·70) | <0·001 |
|  | 18-27 (vs -7) | 1·85 (1·59, 2·16) | <0·001 |
|  | -28 (vs -7) | 2·37 (1·99, 2·83) | <0·001 |
| Admitting department | |  |  |
|  | Medical department (vs Surgical department) | 0·52 (0·46, 0·58) | <0·001 |
| Total case volume of sepsis treatment | |  |  |
|  | Q1 ≤ 235 | 1 |  |
|  | 236 ≤ Q2 ≤ 710 | 0·95 (0·86, 1·06) | 0·360 |
|  | 710 ≤ Q3 ≤ 1743 | 0·83 (0·74, 0·94) | 0·003 |
|  | Q4 ≥ 1743 | 0·42 (0·34, 0·51) | <0·001 |
| CRRT use | | 1·16 (0·94, 1·44) | 0·170 |
| Vasopressor use | | 1·13 (1·00, 1·28) | 0·043 |
| ECMO support | | 1·12 (0·50, 2·52) | 0·790 |
| Mechanical ventilator support | | 2·02 (1·77, 2·30) | <0·001 |
| ICU admission | | 0·90 (0·79, 1·02) | 0·097 |
| Total number of hospital admission for sepsis | |  |  |
|  | 1 | 1 |  |
|  | 2-3 | 1·08 (0·98, 1·19) | 0·110 |
|  | 4-5 | 1·19 (1·00, 1·42) | 0·052 |
|  | 6-7 | 0·90 (0·65, 1·25) | 0·540 |
|  | ≥ 8 | 0·86 (0·63, 1·16) | 0·320 |
| Concurrent psychiatric illness | |  |  |
|  | Depression | 1·11 (1·00, 1·23) | 0·048 |
|  | Anxiety disorder | 0·92 (0·84, 1·02) | 0·130 |
|  | Substance abuse | 1·05 (0·87, 1·28) | 0·590 |
|  | PTSD | 2·82 (0·86, 9·18) | 0·086 |
|  | Bipolar | 1·16 (1·02, 1·31) | 0·025 |
|  | Schizophrenia or schizophrenic affective disorder | 0·90 (0·74, 1·10) | 0·310 |
|  | Dementia | 1·16 (1·05, 1·28) | 0·004 |
| History of Self-harm or suicidal attempt | | 8·28 (3·95, 17·36) | <0·001 |
| Year of diagnosis of sepsis | |  |  |
|  | 2010 | 1 |  |
|  | 2011 | 0·79 (0·66, 0·95) | 0·014 |
|  | 2012 | 1·01 (0·85, 1·21) | 0·870 |
|  | 2013 | 0·86 (0·72, 1·03) | 0·110 |
|  | 2014 | 0·82 (0·69, 0·99) | 0·036 |
|  | 2015 | 0·77 (0·64, 0·93) | 0·006 |
|  | 2016 | 0·72 (0·59, 0·87) | 0·001 |
|  | 2017 | 0·71 (0·59, 0·86) | <0·001 |
|  | 2018 | 0·66 (0·55, 0·81) | <0·001 |

sHR, subdistribution hazard ratio; CI, confidence interval; CRRT, continuous renal replacement therapy; ECMO, extracorporeal membrane oxygenation; ICU, intensive care unit; PTSD, post-traumatic stress disorder
