# Supplementary material for: Prevalence and risk factors for suicide in patients with sepsis: nationwide cohort study in South Korea
Source: BJPsych Open. 2022 Mar 10;8(2):e61. doi: 10.1192/bjo.2022.19 (PMC8935909; doi:10.1192/bjo.2022.19)
Supplement: Supplementary file 1 [file S2056472422000199sup001.zip › S2056472422000199sup011.docx]

Table S4. Competing risk analyses using the Fine and Gray model for suicide mortality in the female

| Variable | | Death by suicide  sHR (95% CI) | *P*-value |
| --- | --- | --- | --- |
| Age, year | |  |  |
|  | 18-35 | 1 |  |
|  | 36-50 | 1·85 (1·10, 3·11) | 0·020 |
|  | 51-65 | 1·55 (0·94, 2·56) | 0·088 |
|  | 65-80 | 2·36 (1·47, 3·80) | <0·001 |
|  | ≥ 81 | 3·93 (2·45, 6·31) | <0·001 |
| Residence at diagnosis of sepsis | |  |  |
|  | Urban | 1 |  |
|  | Rural | 1·11 (1·00, 1·23) | 0·047 |
| Income level at diagnosis of sepsis | |  |  |
|  | Q1 (Lowest) | 1 |  |
|  | Q2 | 1·00 (0·84, 1·19) | 0·990 |
|  | Q3 | 1·04 (0·89, 1·21) | 0·650 |
|  | Q4 (Highest) | 1·04 (0·91, 1·18) | 0·610 |
|  | Unknown | 1·03 (0·89, 1·20) | 0·680 |
| Charlson comorbidity index | |  |  |
|  | 3-6 (vs -2) | 0·86 (0·75, 1·12) | 0·213 |
|  | 7-9 (vs -2) | 1·12 (1·02 1·21) | 0·013 |
|  | -10 (vs -2) | 1·34 (1·20, 1·47) | <0·001 |
| Elixhauser comorbidity index | |  |  |
|  | 8-17 (vs -7) | 1·18 (1·10, 3·11) | 0·020 |
|  | 18-27 (vs -7) | 1·19 (1·02, 1·38) | 0·030 |
|  | -28 (vs -7) | 1·38 (1·15, 1·65) | 0·001 |
| Admitting department | |  |  |
|  | Medical department (vs Surgical department) | 0·57 (0·51, 0·65) | <0·001 |
| Total case volume of sepsis treatment | |  |  |
|  | Q1 ≤ 235 | 1 |  |
|  | 236 ≤ Q2 ≤ 710 | 1·00 (0·89, 1·11) | 0·930 |
|  | 710 ≤ Q3 ≤ 1743 | 0·93 (0·82, 1·06) | 0·280 |
|  | Q4 ≥ 1743 | 0·53 (0·42, 0·67) | <0·001 |
| CRRT use | | 1·39 (1·06, 1·83) | 0·017 |
| Vasopressor use | | 0·97 (0·84, 1·11) | 0·620 |
| ECMO support | | 2·30 (0·73, 7·18) | 0·150 |
| Mechanical ventilator support | | 2·21 (1·92, 2·54) | <0·001 |
| ICU admission | | 0·97 (0·85, 1·10) | 0·630 |
| Total number of hospital admission for sepsis | |  |  |
|  | 1 | 1 |  |
|  | 2-3 | 0·88 (0·80, 0·98) | 0·021 |
|  | 4-5 | 0·69 (0·55, 0·86) | 0·001 |
|  | 6-7 | 0·68 (0·47, 0·99) | 0·047 |
|  | ≥ 8 | 0·36 (0·22, 0·59) | <0·001 |
| Concurrent psychiatric illness | |  |  |
|  | Depression | 0·93 (0·84, 1·04) | 0·220 |
|  | Anxiety disorder | 0·98 (0·88, 1·08) | 0·650 |
|  | Substance abuse | 1·94 (1·26, 2·97) | 0·002 |
|  | PTSD | 4·68 (1·13, 19·38) | 0·033 |
|  | Bipolar | 1·16 (1·01, 1·33) | 0·034 |
|  | Schizophrenia or schizophrenic affective disorder | 1·08 (0·87, 1·34) | 0·480 |
|  | Dementia | 1·50 (1·36, 1·66) | <0·001 |
| History of Self-harm or suicidal attempt | | 9·45 (3·32, 26·91) | <0·001 |
| Year of diagnosis of sepsis | |  |  |
|  | 2010 | 1 |  |
|  | 2011 | 0·90 (0·73, 1·11) | 0·330 |
|  | 2012 | 1·07 (0·88, 1·31) | 0·480 |
|  | 2013 | 1·24 (1·02, 1·51) | 0·031 |
|  | 2014 | 1·09 (0·89, 1·33) | 0·410 |
|  | 2015 | 0·94 (0·76, 1·16) | 0·560 |
|  | 2016 | 0·87 (0·70, 1·07) | 0·180 |
|  | 2017 | 0·84 (0·68, 1·03) | 0·095 |
|  | 2018 | 0·65 (0·52, 0·81) | <0·001 |

sHR, subdistribution hazard ratio; CI, confidence interval; CRRT, continuous renal replacement therapy; ECMO, extracorporeal membrane oxygenation; ICU, intensive care unit; PTSD, post-traumatic stress disorder
