# Supplementary figures and images for: Prevalence and risk factors for suicide in patients with sepsis: nationwide cohort study in South Korea
Source: BJPsych Open. 2022 Mar 10;8(2):e61. doi: 10.1192/bjo.2022.19 (PMC8935909; doi:10.1192/bjo.2022.19)

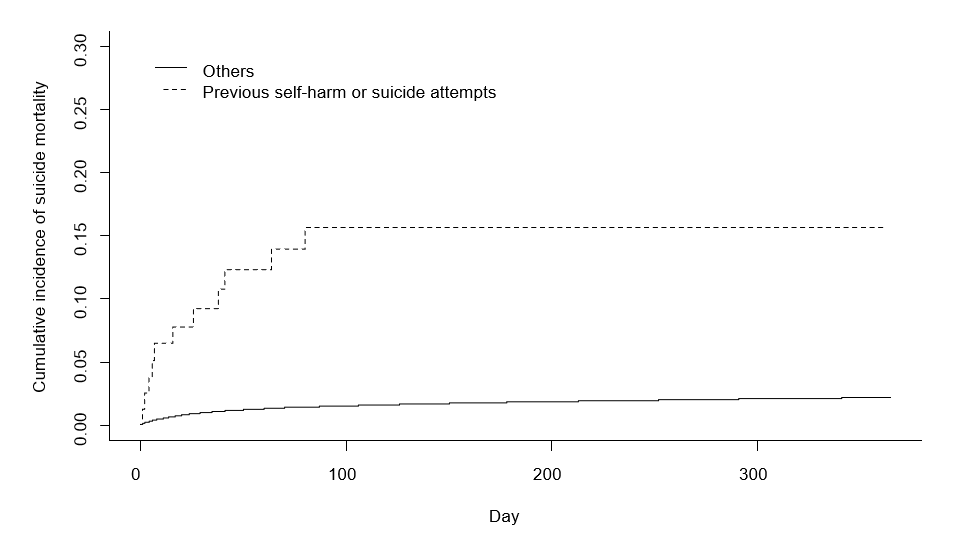

Supplement: Supplementary file 1 [file S2056472422000199sup001.zip › S2056472422000199sup001.tif]

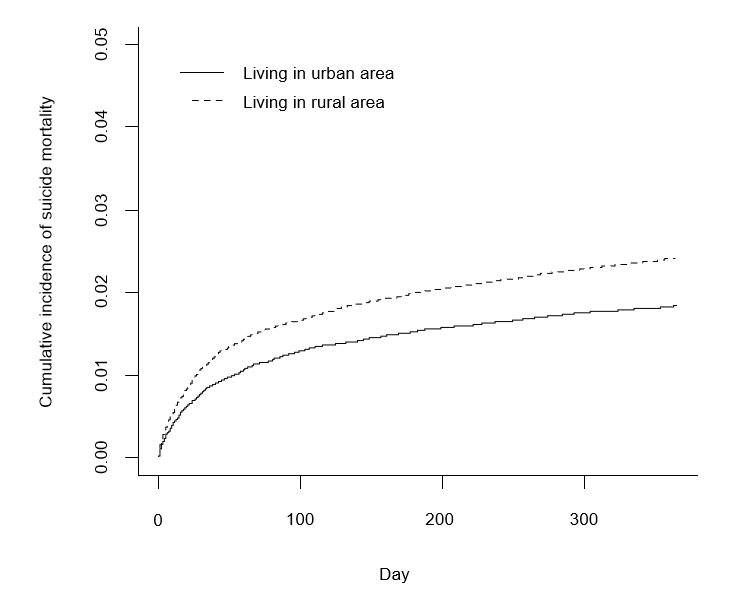

Supplement: Supplementary file 1 [file S2056472422000199sup001.zip › S2056472422000199sup002.tif]

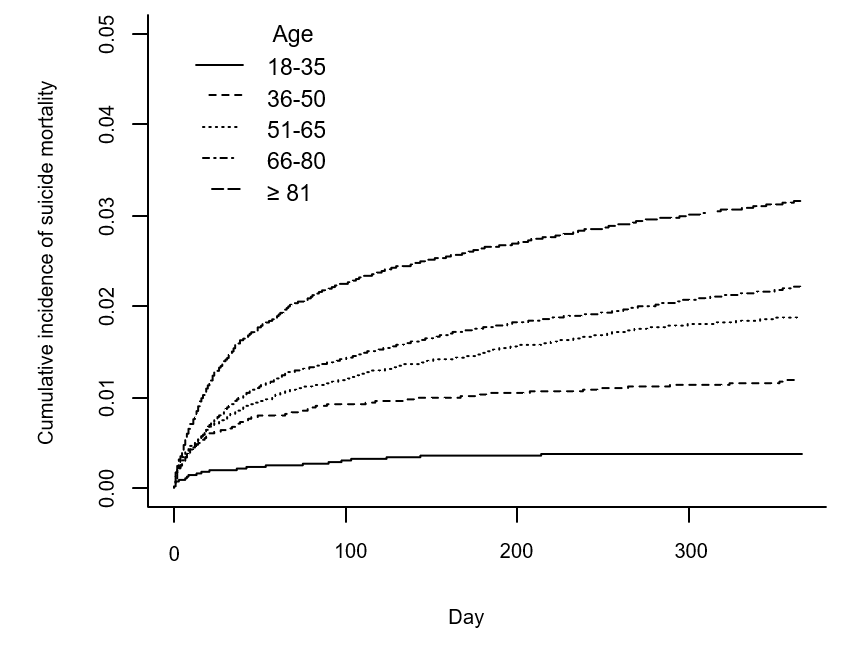

Supplement: Supplementary file 1 [file S2056472422000199sup001.zip › S2056472422000199sup003.tif]

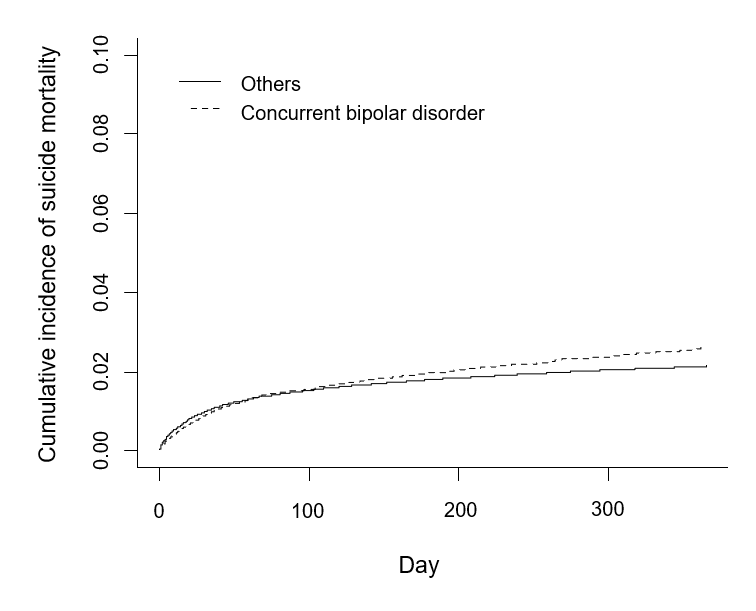

Supplement: Supplementary file 1 [file S2056472422000199sup001.zip › S2056472422000199sup004.tif]

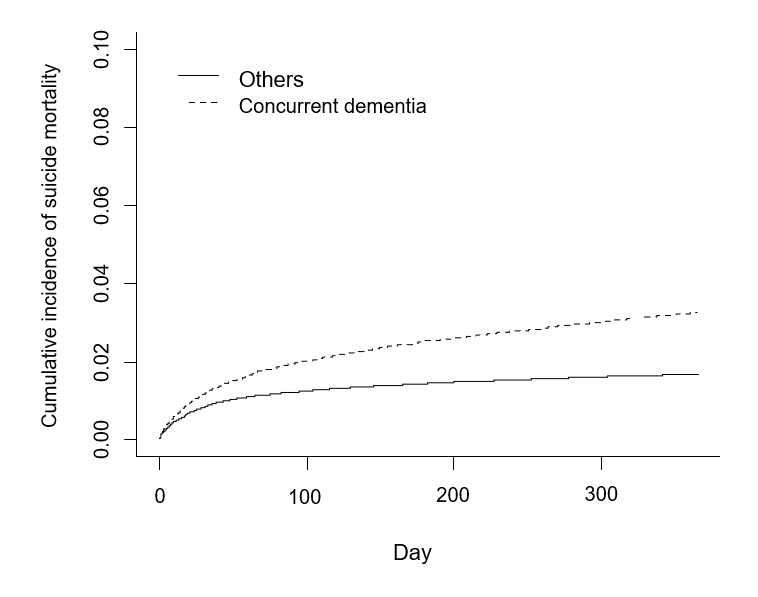

Supplement: Supplementary file 1 [file S2056472422000199sup001.zip › S2056472422000199sup005.tif]

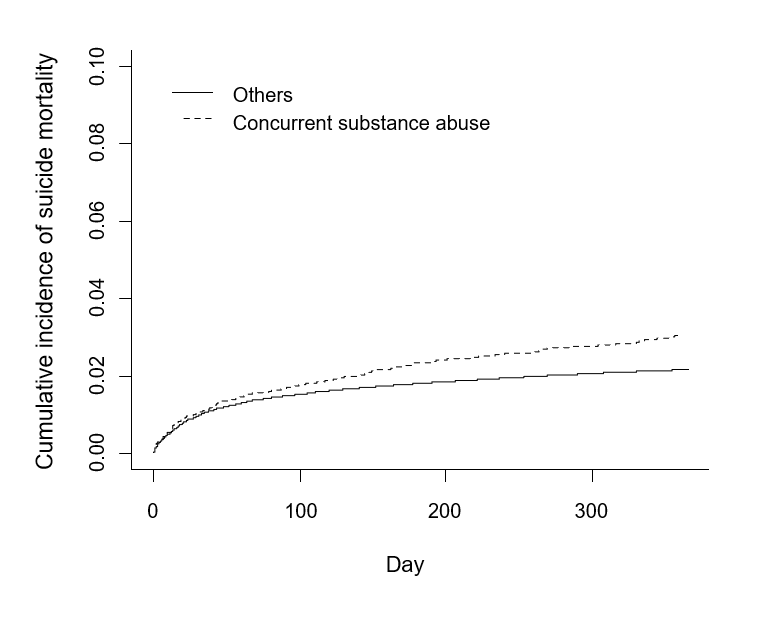

Supplement: Supplementary file 1 [file S2056472422000199sup001.zip › S2056472422000199sup006.tif]

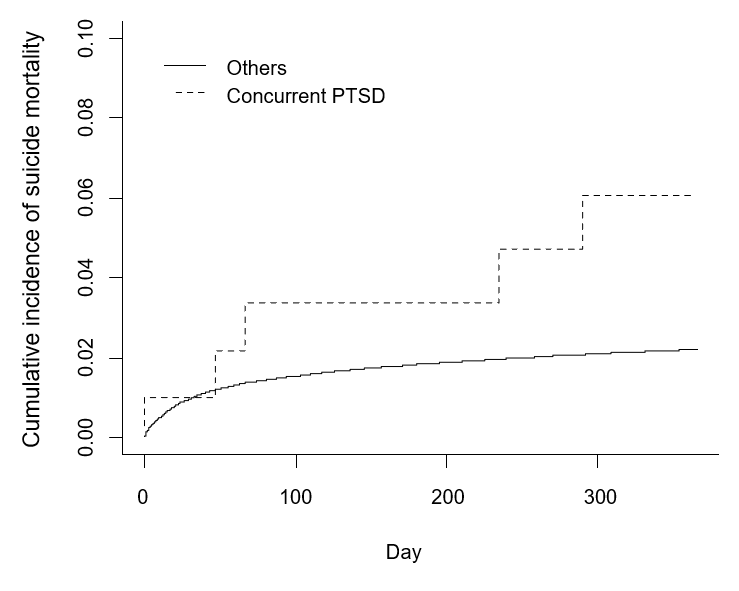

Supplement: Supplementary file 1 [file S2056472422000199sup001.zip › S2056472422000199sup007.tif]
